# Nano Revolution: “Tiny tech, big impact: How nanotechnology is driving SDGs progress"

**DOI:** 10.1016/j.heliyon.2024.e31393

**Published:** 2024-05-16

**Authors:** Basma Elzein

**Affiliations:** a Electrical Engineering Department, College of Engineering, University of Business and Technology, Jeddah, 21451, Saudi Arabia; b Sustainable Development Department, Global Council for Tolerance and Peace, Valetta, Malta

**Keywords:** Nanotechnology, UN SDGs, Nanomaterials, Nanoscience, Nanosensors, Nanoparticles, Nanowires, Nanowalls

## Abstract

Nanotechnology has emerged as a powerful tool in addressing global challenges and advancing sustainable development. By manipulating materials at the nanoscale, researchers have unlocked new possibilities in various fields, including energy, healthcare, agriculture, construction, transportation, and environmental conservation. This paper explores the potential of nanotechnology and nanostructures in contributing to the achievement of the United Nations (UN) Sustainable Development Goals (SDGs) by improving energy efficiency and energy conversion, leading to a more sustainable and clean energy future, improving water purification processes, enabling access to clean drinking water for communities, enabling targeted drug delivery systems, early disease detection, and personalized medicine, thus revolutionizing healthcare, improving crop yields, efficient nutrient delivery systems, pest control mechanisms, and many other areas, therefore addressing food security issues. It also highlights the potential of nanomaterials in environmental remediation and pollution control.

Therefore, by understanding and harnessing nanotechnology's potential, policymakers, researchers, and stakeholders can work together toward a more sustainable future by achieving the 17 UN SDGs.

## 1 Introduction

### 1.1 UN sustainable development goals

**Fig. 1:**
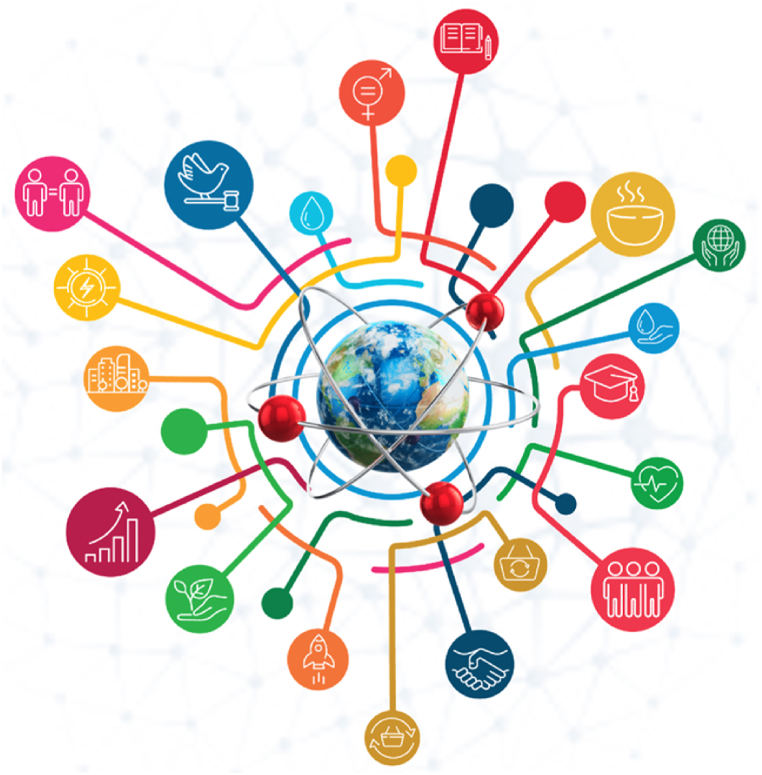
UN Sustainable Development Goals and Nanotechnology (no poverty; zero hunger; good health and well-being; quality education; gender equality; clean water and sanitation; affordable and clean energy; decent work and economic growth; industry, innovation and infrastructure; reduced inequalities; sustainable cities and communities; responsible consumption and production; climate action; life below water; life on land; peace, justice, and strong institutions; and partnerships for the goals).

The globe is today confronted with numerous difficulties that have negative consequences, such as biodiversity loss, deforestation, reckless waste disposal, air pollution, water insecurity, toxic loads, plastic pollution, global warming, and climate change. Social stressors cause illness, limited access to healthcare, low education, and inadequate skill development in various areas, all of which contribute to expanding inequities. Civilization's destiny is uncertain and on a dangerous path. In 2015, the United Nations General Assembly approved a set of 17 goals (Fig. 1) to address these global concerns. In 2017, the United Nations issued an urgent call to action for all countries in its Sustainable Development Goals, recognizing that ending poverty and other deprivations go hand in hand with improving health, the environment, and the economy to reduce inequality in all areas.

The UN SDGs framework is a global initiative consisting of 17 interlinked and universally applicable goals adopted by UN member states as part of the 2030 agenda for sustainable development. They serve as a shared blueprint for addressing global challenges and achieving a more sustainable, equitable, and prosperous future by 2030.

Paragraph 70 of the 2030 Agenda reads: *“The Technology Facilitation Mechanism will be based on a multi-stakeholder collaboration between Member States, civil society, private sector, the scientific community, United Nations entities, and other stakeholders and will be composed of a United Nations Interagency Task Team on Science, Technology, and Innovation for the SDGs, a collaborative Multistakeholder Forum on Science, Technology, and Innovation for the SDGs and an on-line platform.”*

Since the inception of the Sustainable Development Goals, numerous favorable advancements have occurred. Nations are actively integrating these goals into their national plans and strategies, and a considerable number have established coordinating frameworks to ensure a unified and effective implementation. Nevertheless, despite the initial endeavors, progress toward attaining most of the targets encompassed by the goals is not on the anticipated trajectory. However, there is a reason for hope. Science plays a pivotal role in pursuing the Sustainable Development Goals (SDGs), serving as a catalyst for innovation, problem-solving, and evidence-based decision-making. The intricate challenges addressed by the SDGs, spanning poverty alleviation, healthcare improvement, environmental sustainability, and beyond, necessitate a robust scientific foundation. Through scientific research and advancements, we gain insights into complex issues, develop sustainable technologies, and formulate effective policies. Scientific methods enable us to monitor progress, identify areas for intervention, and assess the impact of various initiatives.

Moreover, science fosters collaboration and knowledge-sharing among nations, paving the way for global partnerships essential for achieving the interconnected and ambitious targets of the SDGs. From developing renewable energy solutions to designing resilient infrastructure, science provides the tools and knowledge required to build a more equitable, sustainable, and resilient future for all. In essence, the importance of science in achieving the SDGs lies in its capacity to drive informed, evidence-driven actions that are essential for addressing the multifaceted challenges facing our world.

While each of the Sustainable Development Goals (SDGs) encompasses a broad range of issues, including social, economic, and environmental dimensions, here's a brief overview of each goal from a scientific perspective, presented in Table 1.

**Table 1:** Scientific insight of the 17 SDGs.

Goal 1: No Poverty	Poverty has complex roots, and addressing it requires interdisciplinary research. Scientists study economic systems, social structures, and policies to understand poverty dynamics and develop evidence-based strategies for poverty reduction.
Goal 2: Zero Hunger	Agricultural science plays a crucial role in achieving food security. Research focuses on improving crop yields, sustainable farming practices, and developing resilient crops that can withstand climate change impacts.
Goal 3: Good Health and Well-Being	Medical and health sciences contribute to understanding diseases, developing vaccines, and improving healthcare systems. Epidemiology and public health research inform strategies for preventing and treating diseases.
Goal 4: Quality Education	Educational research explores effective teaching methods, curriculum development, and the impact of education on individual and societal well-being. Science, technology, engineering, and mathematics (STEM) education is particularly crucial.
Goal 5: Gender Equality	Gender studies and social sciences provide insights into the root causes of gender inequality. Research helps identify barriers and effective interventions to promote gender equality in various sectors.
Goal 6: Clean Water and Sanitation	Environmental science, hydrology, and engineering are essential for investigating water availability, quality, and sanitation issues. Sustainable water management strategies are developed through scientific research.
Goal 7: Affordable and Clean Energy	Energy science and engineering contribute to the development of renewable energy sources, energy efficiency technologies, and sustainable energy policies. Research focuses on making clean energy accessible and affordable.
Goal 8: Decent Work and Economic Growth	Economics, sociology, and labor studies play a role in understanding economic systems, labor markets, and policies that promote decent work. Research informs strategies for inclusive economic growth.
Goal 9: Industry, Innovation, and Infrastructure	Science, technology, and engineering drive innovation and industrial development. Research in these fields contributes to sustainable infrastructure, technological advancements, and economic progress.
Goal 10: Reduced Inequality	Social sciences, economics, and sociology investigate the drivers of inequality. Research informs policies aimed at reducing disparities in income, education, and access to opportunities.
Goal 11: Sustainable Cities and Communities	Urban planning, environmental science, and architecture contribute to creating sustainable cities. Research explores ways to manage urban growth, reduce environmental impact, and enhance livability.
Goal 12: Responsible Consumption and Production	Environmental science and sustainability research examine resource consumption patterns, waste management, and the environmental impact of production. Scientific findings guide policies for responsible consumption.
Goal 13: Climate Action	Climate science and environmental research provide insights into climate change causes and impacts. Scientists develop mitigation and adaptation strategies to address the challenges posed by climate change.
Goal 14: Life Below Water	Marine biology, oceanography, and environmental science contribute to understanding marine ecosystems. Scientific research informs conservation efforts and the sustainable management of marine resources.
Goal 15: Life on Land	Ecology, forestry, and biodiversity studies contribute to understanding terrestrial ecosystems. Scientific research guides conservation efforts and sustainable land management practices.
Goal 16: Peace, Justice, and Strong Institutions	Political science, law, and criminology contribute to understanding conflict, promoting justice, and strengthening institutions. Research informs strategies for building peaceful and just societies.
Goal 17: Partnerships for the Goals	Interdisciplinary research is critical for effective partnerships. Scientific collaborations provide evidence-based approaches to address global challenges and achieve sustainable development goals through international cooperation.

The scientific perspective on the SDGs emphasizes the importance of interdisciplinary research, evidence-based policies, and technological innovations to address humanity's complex challenges in pursuing sustainable development.

### 1.2 Nanotechnology

To achieve these 17 goals, it is time to recognize the crucial role of science and technology in tackling and solving the current challenges addressed by these goals.

As an emerging technology, nanotechnology (the science and technology at the atomic level) has the potential to significantly contribute to achieving these goals, since it will deliver disruptive, game-changing discoveries and innovations that benefit our society, environment, and planet.

Nanotechnology, the science of altering matter on an atomic scale (10^−9^ m), has evolved rapidly in recent years as a result of its prospective applications in disciplines such as medicine, electronics, and energy. It all started with Richard Feynman [1], a physicist, who initially presented the concept of nanotechnology in his famous lecture "There's Plenty of Room at the Bottom" in 1959. In this lecture, Feynman highlighted the prospect of manipulating and directing individual atoms and molecules to build new materials and gadgets. This was followed in 1974 by Norio Taniguchi [2], a Japanese physicist, who coined the word “nanotechnology" in a paper entitled “On the Basic Concept of ‘Nanotechnology’”. In this study, Taniguchi highlighted the idea of producing new materials and gadgets by controlling matter on a nanoscale. His work popularized the term “nanotechnology" and paved the way for future study in this sector. Other physicists, Gerd Binnig and Heinrich Rohrer [3], devised the Scanning Tunneling Microscope (STM) in 1981, allowing scientists to directly examine and manipulate individual atoms. This discovery sparked a boom in interest in nanotechnology research and development. In 1986, researcher Eric Drexler [4] released “Engines of Creation: The Coming Era of Nanotechnology," in which he predicted that nanotechnology would be utilized to manufacture new materials, machines, and even entire cities. Since then, nanotechnology has advanced significantly, bringing new discoveries and developments every year. Don Eigler [5], who utilized the STM to spell out “IBM" using individual xenon atoms, and Sumio [6], who discovered carbon nanotubes in 1991, are two notable persons who have contributed to the advancement of nanotechnology. In 1996, Richard Smalley received the Nobel Prize in Chemistry for his work on fullerenes, which are spherical molecules composed of carbon atoms [7].

Since then, scientists have studied Nanotechnology and found that working at the nanoscale is all about mimicking nature in developing self-cleaning surfaces, photosynthesis, high tensile strength and elasticity, and biological processes in our bodies. Inspired by the lotus leaf, which repels water due to its nano-scale structures, scientists have created superhydrophobic coatings that can be applied to various surfaces to repel water and prevent dirt and contaminants from sticking, making them self-cleaning [8,9]. Such surfaces find applications in industries such as automotive, aerospace, and architecture. Furthermore, Photosynthesis is nature's way of converting sunlight into chemical energy. Scientists are working on replicating this process by designing nanomaterials that can capture sunlight and convert it into useable energy [10], such as hydrogen fuel. These artificial photosynthetic systems have the potential to revolutionize renewable energy production by providing a clean and sustainable source of fuel. In addition, Spider silk is known for its exceptional mechanical properties, including high tensile strength and elasticity. Scientists have developed synthetic spider silk using nanofabrication techniques [11,12] that mimic the structure and properties of natural spider silk. This synthetic silk has potential applications in various fields, including textiles, medicine (such as tissue engineering), and even bulletproof vests. Furthermore, nanoparticles can be designed to encapsulate drugs and deliver them to specific targets in a controlled manner, mimicking the behavior of cells, such as crossing biological barriers or responding to specific stimuli. This approach allows for more targeted and efficient drug delivery, minimizing side effects and improving therapeutic outcomes.

### 1.3 Research methodology

This review paper presents a comprehensive exploration of the recent advancements and diverse applications of Nanotechnology in terms of Nanomaterials and nanostructures within the context of the United Nations' 17 Sustainable Development Goals (SDGs).

Through an exhaustive analysis of existing research literature, coupled with data collection from varied sources, key findings have been synthesized and organized into a coherent framework. This framework illustrates how nanotechnology can play a pivotal role in advancing the objectives outlined in the UN SDGs. By harnessing insights derived from scientific research and innovative technologies, this paper contributes to the ongoing dialogue surrounding sustainable development. Moreover, it underscores the transformative potential of nanotechnology in addressing complex global challenges, thereby fostering progress toward a more equitable and sustainable future.

The research methodology began with an extensive literature review, delving into the depths of academic and scientific publications focused on nanotechnology and its numerous applications across various sectors relevant to the UN Sustainable Development Goals (SDGs). The research articles, review papers, conference proceedings, and reports published in reputable journals, databases, and repositories were carefully selected, specifically the ones that shed light on how nanotechnology intersects with the UN SDGs.

Based on the relevant literature, the next step involved gathering empirical findings, theoretical frameworks, experimental results, and illuminating case studies that depicted the impact of nanotechnology on sustainable development goals. These valuable insights were compiled into the applications of different nanostructures and nanomaterials in addressing each of the 17 SDGs from these designated research papers and publications.

As the data accumulated, the collected information was synthesized, analyzed, and organized by identifying key findings, recognizing emerging trends, and extracting profound insights that elucidated the role of nanotechnology in advancing the SDGs. A thorough analysis of the literature was conducted to understand the complex ways in which nanotechnology helps to achieve sustainable development goals. This includes exploring scientific advancements, policy implications, and social effects.

One of the pivotal phases of the research involved mapping nanotechnology applications to the UN SDGs. The latest findings and applications identified in the literature review were aligned with each of the 17 Sustainable Development Goals, drawing connections between specific nanotechnological advancements and their contributions to addressing the targets and indicators associated with each goal. Through this process, the interdisciplinary nature of nanotechnology and its remarkable potential to influence the achievement of the UN SDGs through many innovative applications were highlighted.

As the research journey progressed, a comprehensive framework encapsulating nanotechnology's transformative potential in driving progress toward the UN SDGs was developed. This framework served as a structured blueprint, illustrating the alignment between specific nanotechnological interventions and the overarching goals of sustainable development. By integrating the synthesized findings into this framework, the research aimed to provide a holistic understanding of how nanotechnology can catalyze progress toward a more equitable and sustainable future for all.

It is worth mentioning that this review paper represents a pioneering effort to bridge the gap between Nanotechnology findings and the United Nations' 17 SDGs.

To the best of our knowledge, no previous study has systematically linked nanotechnology's findings and applications to all 17 SDGs, illustrating the connections between them. This unique work fills a critical knowledge gap and provides valuable insights into nanotechnology's potential contributions toward achieving global sustainability targets. By unveiling these connections, this paper aims to inspire further interdisciplinary research and collaboration, driving innovation towards a more prosperous and sustainable future for all.

## 2 Nanotechnology and the 17 SDGs

Nanotechnology is now an interdisciplinary field bringing together researchers from physics, chemistry, biology, engineering, and materials science to transform numerous industries and open up new avenues for innovation and discovery, thus developing communication, negotiation, problem-solving, analytical thinking, and many other soft skills. Through the development of smart materials and connected devices, Nano is making an impact in different sectors such as energy, environmental protection, resource management, and healthcare. Summarized applications of different nanostructures are presented in Table 2....

**Table 2:** Applications of the different nanostructures.

Nanomaterial	Description	Applications
0D nanostructures (Quantum Dots Nanoparticules)	Zero-dimensional nanostructures, also known as zero-dimensional nanomaterials or quantum dots, are nanoscale materials that have a dimensionality of zero. It can be presented in the form of nanocrystals, Quantum dots, Nanoparticles, and fullerenes. Nanocrystals are small crystalline structures with dimensions in the nanometer range. They can be composed of various materials such as metals, metal oxides, or semiconductors. Nanocrystals exhibit size-dependent properties Quantum dots are considered semiconductor nanocrystals with a size-dependent bandgap, exhibiting unique optical and electronic properties due to quantum confinement effects. Quantum dots can emit light at specific wavelengths when excited, generating multiple electrons. Nanoparticles are also in the size of nanometers and made from various materials like metals (e.g., gold nanoparticles), metal oxides (e.g., titanium dioxide nanoparticles), or polymers. Fullerenes are spherical carbon molecules consisting of carbon atoms arranged in a cage-like structure. The most well-known fullerene is Buckminsterfullerene (C60), which has 60 carbon atoms arranged in a soccer ball-like shape, and they are characterized by their unique electronic properties [[13], [14], [15], [16], [17], [18], [19], [20], [21]]. (Fig. 2)	Solar Cells, Imaging, LEDs, drug delivery, Air purification, water treatment, Self-cleaning surfaces, Paintings, Quantum computing, Soil, nutrients
1D nanostructures (Nanowires, Carbon Nanotubes)	One-dimensional nanostructures are nanoscale materials that possess extended dimensions in one direction while being significantly smaller in the other two dimensions. These structures exhibit unique properties and behaviors due to their confined geometry, they possess large surface areas, high aspect ratios, and elongated structures, making them suitable for the electron transfer process. They are present in the form of Nanowires, nanotubes, nanorods, and nanobelts. Nanowires are considered cylindrical, hexagonal, cubical, and triangular structures with diameters typically ranging from a few nanometers to a few hundred nanometers, while their length can extend up to several micrometers or even longer. They can be made from various materials, including metals, semiconductors, or oxides. Nanotubes are hollow structures composed of carbon or other materials such as boron nitride or metal oxides. Carbon nanotubes (CNTs) are the most well-known and extensively studied type of nanotubes. They exhibit exceptional mechanical strength, electrical conductivity, and thermal properties. Nanorods are elongated structures like nanowires with larger diameters in the nanometer range and lengths typically several times larger than their diameter. They can be made from various materials like metals, semiconductors, or oxides. Nanorods exhibit anisotropic properties due to their elongated shape. Nanobelts are thin ribbon-like structures with widths in the nanometer range and lengths typically much larger than their width. They can be made from various materials like semiconductors, oxides, or metals. Nanobelts exhibit unique electrical, optical, and mechanical properties [[22], [23], [24], [25], [26], [27], [28], [29], [30], [31]]. (Fig. 3)	Solar Cells, CO_2_ Capturing, water purification, Batteries, Cancer Treatment, photosynthesis, displays, Chemical detections, wearable devices, Infrared (IR) and radiation detection
2D nanostructures (Nanowalls, Nanosheets, thin films)	Two-dimensional (2D) nanostructures are materials or structures that have a thickness or height of only a few atomic or molecular layers, while their other dimensions can extend to macroscopic scales. These nanostructures possess unique properties and behaviors due to their reduced dimensionality. In a two-dimensional nanostructure, the atoms or molecules are arranged in a specific pattern within the two-dimensional plane. This arrangement can be regular, such as in a crystal lattice, or irregular, depending on the material and fabrication method. One of the most well-known examples of a two-dimensional nanostructure is graphene. Graphene is composed of a single layer of carbon atoms arranged in a hexagonal lattice. It is an excellent conductor of electricity and heat and possesses exceptional mechanical strength [[32], [33], [34], [35], [36], [37], [38]].(Fig. 4)	Batteries, supercapacitors, sensors, tissue engineering, energy conversion, membranes, water purification, self-cleaning and self-healing surfaces, sanitation, coatings, drug delivery, Electronic devices, and sensors

**Fig. 2:**
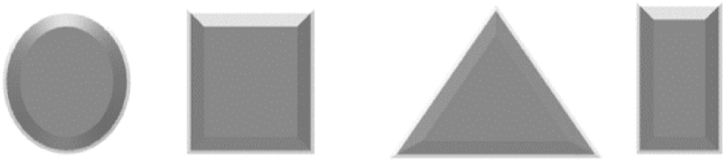
Illustration of zero-dimensional nanostructures.

**Fig. 3:**
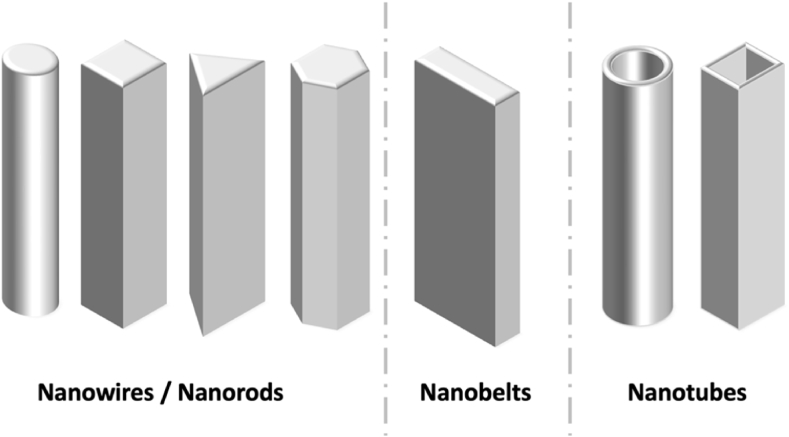
Illustration of one-Dimensional Nanostructures.

**Fig. 4:**
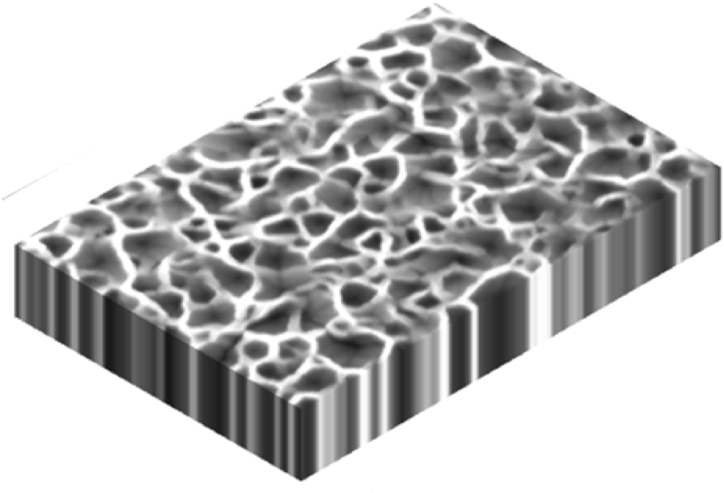
Illustration of two-Dimensional Nanostructures [39].

Thus, by drawing inspiration from natural systems and working at the atomic level, scientists can achieve the UN 17 Sustainable Development Goals. Nanotechnology is diverse and holds great potential for solving real-world challenges [40] and transforming many parts of human life.

**Fig. 5:**
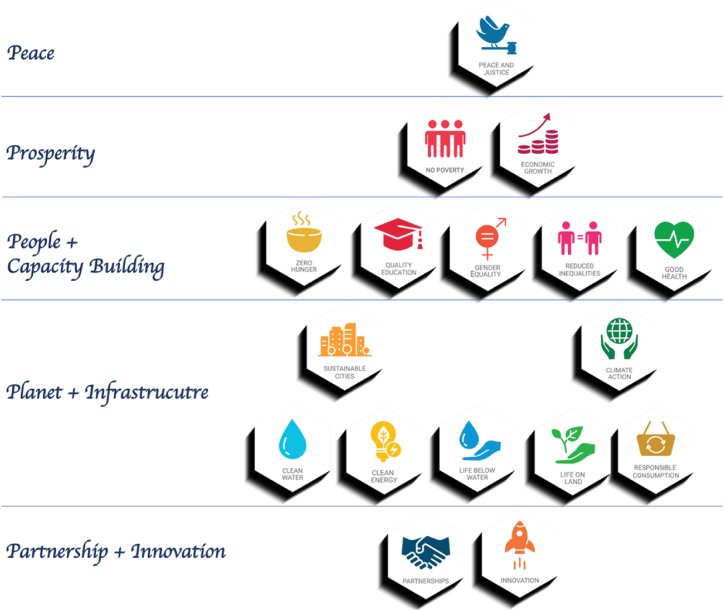
Interlinked SDGs.

It is important to note that these 17 goals are interrelated (Fig. 5), and achieving one will result in achieving the others. The world's utmost goal is to bring about world peace, which can be achieved by fostering prosperity (by achieving no poverty and economic growth goals), which is based on supporting people and building their capacity (by achieving Zero hunger, quality of education, gender equality, reducing inequality, and good health goals). People and capacity building is based on protecting the planet and building the infrastructure (by achieving clean water, clean energy, life on land, life underwater, consumption and production, as well as building sustainable cities and addressing climate change issues goals). Gathering well-experienced people, establishing national and international partnerships, and working on innovative solutions using science and technology to meet all the above-mentioned goals can be achieved.

Acknowledging the crucial role of science and technology and aligning with the overarching targets and indicators outlined in Table 3 for the 17 Sustainable Development Goals (SDGs), this paper aims to delve into the application of nanotechnology in advancing these goals. The comprehensive framework established by these goals, along with their specific targets and indicators, serves as a roadmap for measuring and addressing diverse facets of sustainable development. This framework plays a pivotal role in steering worldwide initiatives aimed at enhancing the quality of life for individuals and fostering environmental stewardship.

**Table 3:** Overall targets and indicators for the 17 SDGs.

Goals	Overall Target	Overall Indicator
Goal 1: no Poverty	Eradicate extreme poverty, reduce poverty rates, and promote social protection systems.	Percentage of the population living below the international poverty line, social protection coverage.
Goal 2: Zero Hunger	End hunger, achieve food security, improve nutrition, and promote sustainable agriculture.	Prevalence of undernourishment, stunting, wasting, agricultural productivity, and food security.
Goal 3: Good Health and well-Being	Ensure healthy lives and promote well-being for all at all ages.	Maternal mortality rate, child mortality rate, disease prevalence, access to healthcare, and life expectancy.
Goal 4: Quality Education	Ensure inclusive and equitable quality education and promote lifelong learning opportunities for all.	Literacy rates, school enrollment, and education quality measures.
Goal 5: Gender Equality	Achieve gender equality and empower all women and girls.	Gender wage gap, political representation, and access to education and healthcare.
Goal 6: Clean Water and Sanitation	Ensure availability and sustainable management of water and sanitation for all.	Access to clean water sources, sanitation facilities, and water quality.
Goal 7: Affordable and Clean Energy	Ensure access to affordable, reliable, sustainable, and modern energy.	Access to electricity, use of renewable energy sources, and energy efficiency.
Goal 8: Decent Work and Economic Growth	Promote sustained, inclusive, and sustainable economic growth, full and productive employment, and decent work for all.	Employment rates, GDP growth, and labor market indicators.
Goal 9: Industry, Innovation, and Infrastructure	Build resilient infrastructure, promote inclusive and sustainable industrialization, and foster innovation.	Infrastructure development, research and development expenditure, and technological progress.
Goal 10: Reduced Inequality	Reduce inequality within and among countries.	Income inequality, social inclusion, and economic disparities.
Goal 11: Sustainable Cities and Communities	Make cities and human settlements inclusive, safe, resilient, and sustainable.	Urbanization, access to adequate housing, and sustainable urban development.
Goal 12: Responsible Consumption and Production	Ensure sustainable consumption and production patterns.	Resource efficiency, waste generation, and sustainable practices.
Goal 13: Climate Action	Take urgent action to combat climate change and its impacts.	Greenhouse gas emissions, climate adaptation measures, and climate-related policies.
Goal 14: Life Below Water	Conserve and sustainably use the oceans, seas, and marine resources for sustainable development.	Marine biodiversity, overfishing, and marine conservation efforts.
Goal 15: Life on Land	Protect, restore, and promote sustainable use of terrestrial ecosystems, sustainably manage forests, combat desertification, halt and reverse land degradation, and halt biodiversity loss.	Forest cover, land degradation, and wildlife conservation.
Goal 16: Peace, Justice, and Strong Institutions	Promote peaceful and inclusive societies for sustainable development, provide access to justice for all, and build effective, accountable, and inclusive institutions.	Rule of law, political stability, and corruption indices.
Goal 17: Partnerships for the Goals	Strengthen the means of implementation and revitalize the global partnership for sustainable development.	Official development assistance, global cooperation, and partnerships for development.

### 2.1 Partnerships for the goals (Goal 17)

By promoting international partnerships, knowledge sharing, and technology transfer, nanotechnology can accelerate scientific advancements and technological innovations. Collaborative efforts can lead to breakthroughs in nanomaterial synthesis, characterization techniques, and device fabrication, benefiting multiple sectors such as healthcare, energy, and environmental sustainability. Furthermore, nanotechnology can facilitate partnerships between academia, industry, and government agencies. Collaborations between these sectors are essential for translating nanotechnology research into practical applications and commercial products. Nanotechnology-based startups and spin-off companies can emerge from such partnerships, driving economic growth and job creation.

**Fig. 6:**
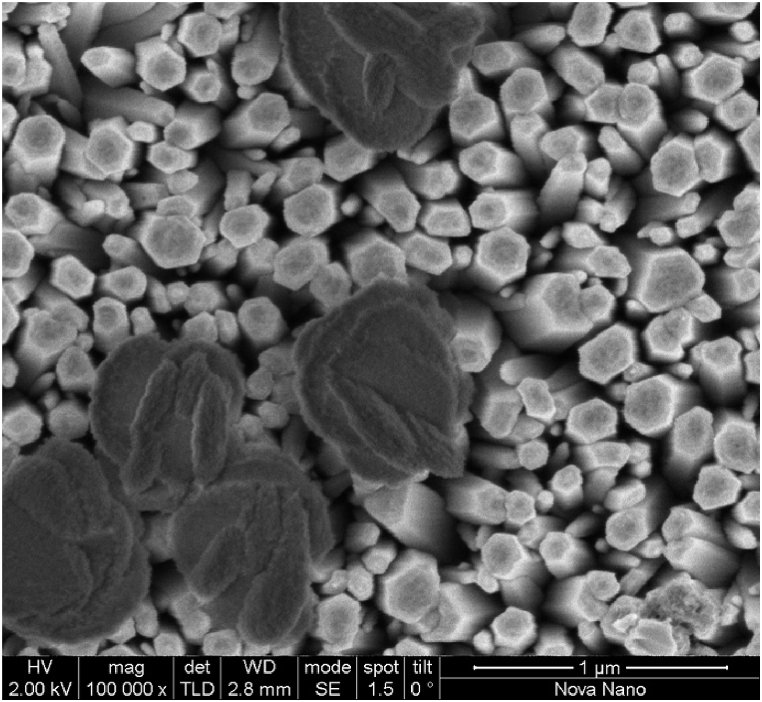
Scanning Electron Microscope (SEM) image: Lead Sulfide (PbS) Nanoparticles deposited over Zinc Oxide (ZnO) Nanowire.

**Fig. 7:**
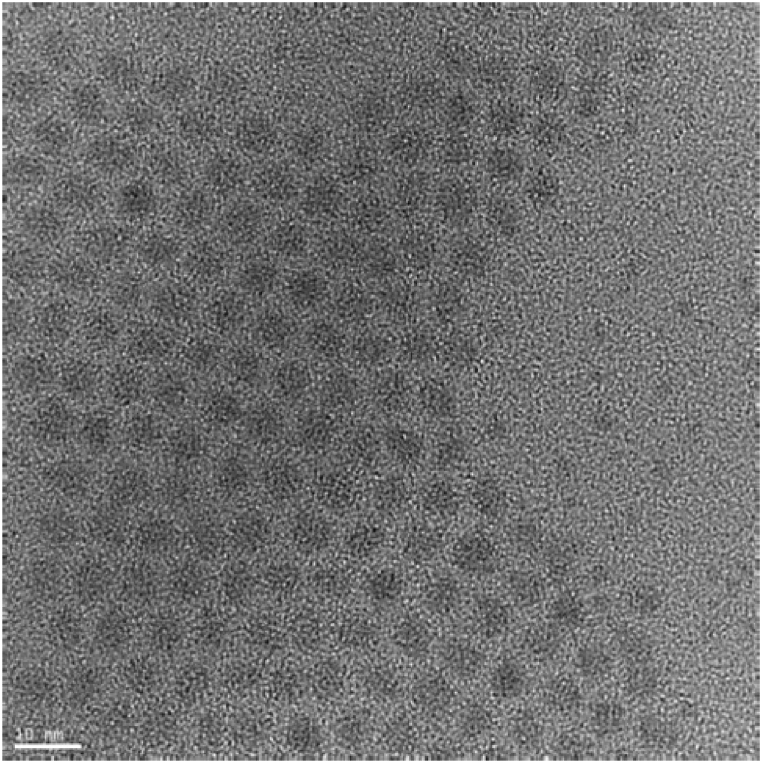
Transmission Electron Microscope (TEM) image of Quantum Dots [29].

Additionally, public-private partnerships can support the development of nanotechnology infrastructure and facilities, enabling more efficient research and development processes. Nanotechnology also has the potential to promote global partnerships for sustainable manufacturing practices. By developing environmentally friendly nanomaterials and manufacturing processes, countries can collaborate to reduce their carbon footprint and minimize the environmental impact of industrial activities. Nanotechnology-enabled solutions such as energy-efficient nanoparticles (Fig. 6, Fig. 7) or eco-friendly coatings can contribute to sustainable production practices across different industries. Moreover, nanotechnology can facilitate partnerships for capacity building in developing countries. Access to advanced technologies and scientific knowledge is often limited in these regions. By promoting technology transfer and knowledge exchange programs, developed countries can support the development of nanotechnology capabilities in developing nations. This can empower local researchers and entrepreneurs to address their specific societal challenges, such as healthcare, agriculture, or clean water access, using nanotechnology-based solutions.

Examples of International Collaborations related to Nanotechnology.

- European Union's Horizon 2020 Program: The Horizon 2020 program has funded various nanotechnology-related projects involving collaboration between multiple European countries. Examples include the Graphene Flagship project, which focuses on developing graphene-based technologies.
- International Nanotechnology Laboratory (INL): The INL, based in Portugal, is an international research organization that collaborates with multiple countries to advance nanotechnology research. It serves as a hub for interdisciplinary research and innovation.
- Global Nanotechnology Network (GNN): The GNN facilitates collaboration among researchers and institutions worldwide. It supports projects that aim to address global challenges through nanotechnology, fostering international cooperation.
- U.S.-China Clean Energy Research Center on Nanotechnology: The U.S. and China have collaborated on nanotechnology research through initiatives such as the Clean Energy Research Center. These collaborations focus on applications of nanotechnology in clean energy and environmental sustainability.
- Nanomedicine European Technology Platform (ETP): Nanomedicine ETP involves collaboration among European countries to advance research and development in nanomedicine. This platform aims to accelerate the translation of nanotechnologies into clinical applications.
- Japan-EU Nanotechnology Symposium: Events such as the Japan-EU Nanotechnology Symposium bring together researchers from Japan and the European Union to discuss advancements in nanotechnology and explore collaborative opportunities.
- Brazilian Nanotechnology National Laboratory (LNNano) Collaborations: LNNano in Brazil collaborates with various international partners on nanotechnology research. Collaborations include joint projects, student exchanges, and participation in international research networks.
- International Consortium for Nanotechnology in Medicine (ICN): The ICN involves collaborative efforts among researchers from different countries to advance nanotechnology applications in medicine. It focuses on developing innovative solutions for healthcare challenges.

### 2.2 Industry, innovation, and infrastructure (Goal 9)

By spurring innovation and enhancing infrastructure, nanotechnology can significantly contribute to the UN SDGs. Nanotechnology is at the forefront of technological innovation. Creating new materials with unique features, such as stronger and more resilient building materials [[41], [42], [43], [44], [45], [46]], can enhance the performance of already-existing infrastructure. Nanotechnology can also advance renewable energy sources and increase energy efficiency [21,[47], [48], [49], [50]], spurring innovation in the energy industry. Furthermore, manufacturing techniques based on nanotechnology [51,52] can increase productivity and decrease waste, resulting in more environmentally friendly and economically viable production techniques. Nanosensors can enhance reliability and safety by identifying potential problems before they become severe and monitoring infrastructure in real time. Finally, waste [[53], [54], [55]] can be reduced, and water treatment [54,56,57] plants can operate more effectively thanks to nanotechnology-based water treatment technologies (elaborated in detail in the following sections).

### 2.3 Clean water and sanitation (Goal 6)

By harnessing nanotechnology, we can revolutionize water treatment and purification, paving the way for universal access to clean water and sanitation. Nanotechnology can help improve water filtration, desalination, purification, and monitoring.

Water pollutants, such as bacteria, viruses, heavy metals, and other contaminants, that are challenging to remove using conventional techniques, can be eliminated from water at the molecular level utilizing nanotechnology-based filters [54,58,59]. Additionally, using nano-porous membranes to filter out salt and other pollutants, seawater can be desalinated [[60], [61], [62], [63], [64]] and converted into potable water. Metal oxide nanoparticles can also filter water by eliminating bacteria and viruses. Silver nanoparticles are particularly good at eliminating hazardous microbes from water. Finally, nano-sensors can be utilized to monitor water quality in real time by identifying impurities and notifying authorities before the situation deteriorates.

Singapore's “NEWater"^1^ project used advanced nanofiltration and reverse osmosis technologies to recycle wastewater into high-purity drinking water. The application of nanotechnology in water treatment not only ensures access to clean water but also conserves water resources and reduces pollution, contributing to water and sanitation goals.

### 2.4 Affordable and clean energy (Goal 7)

**Fig. 8:**
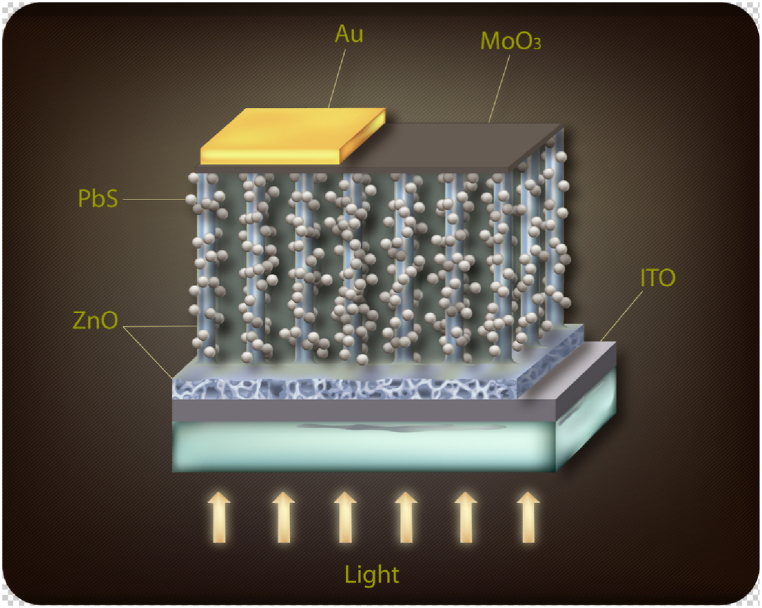
Illustration of quantum dots Sensitized solar cell [29].

**Fig. 9:**
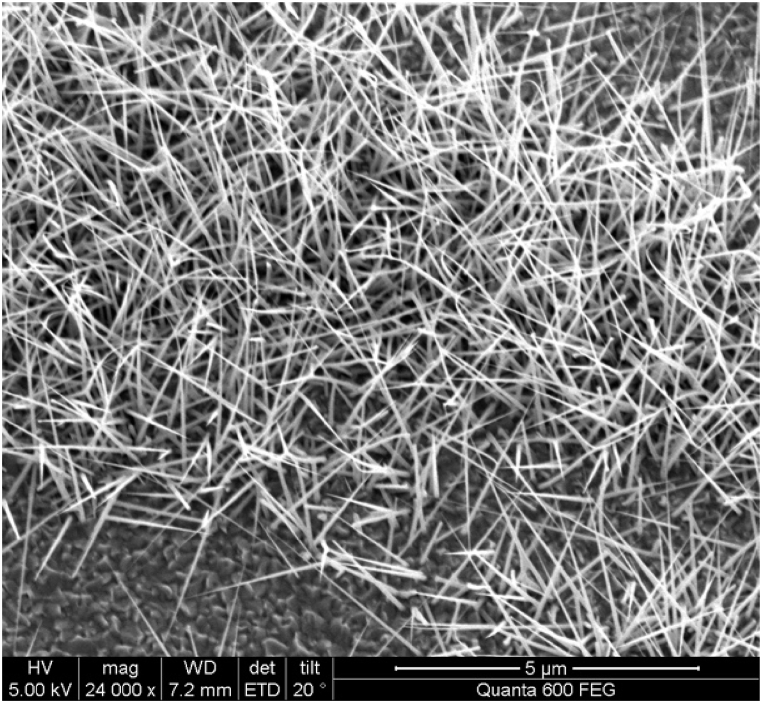
SEM image - ZnO Nanowires.

**Fig. 10:**
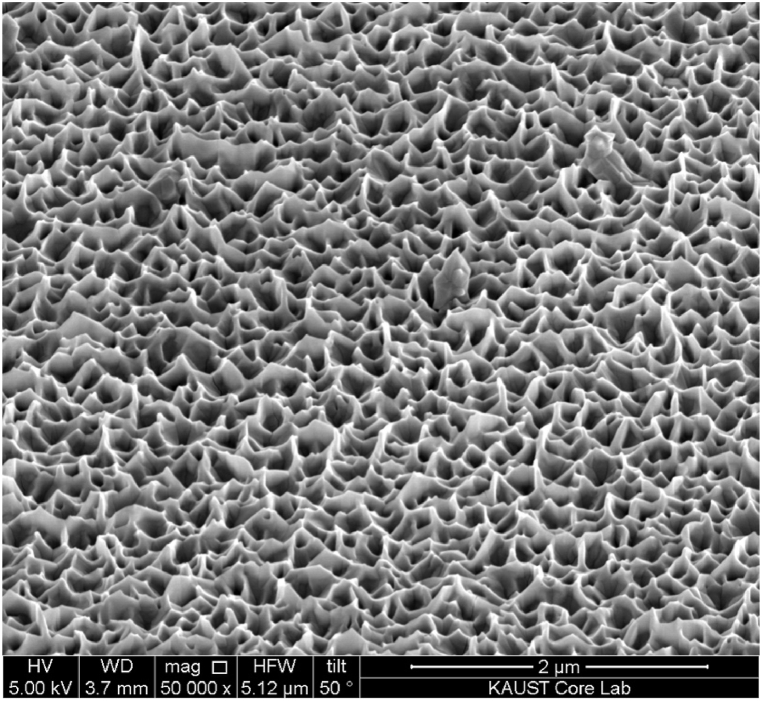
SEM image- ZnO Nanowalls.

Nanotechnology can potentially improve the efficiency and cost-effectiveness of renewable energy sources, such as solar cells [30,37,65,66] (using Zinc oxide Nanowires, and Quantum dots as illustrated in Fig. 8, Fig. 9) and fuel cells [67,68]. Furthermore, nanomaterials can be employed to develop more efficient and durable versions of energy storage devices, such as batteries [34,[69], [70], [71]] and capacitors [37,[72], [73], [74]](using Nanowalls - Fig. 10). Researchers are working today on printed and flexible solar cells, as well as batteries. Nanotechnology can also enhance the energy efficiency of buildings, vehicles, and manufacturing processes, reducing energy consumption and greenhouse gas emissions. In addition, nanotechnology-based sensors [38,[75], [76], [77]] can be used to monitor energy usage in real-time, improving the efficiency of energy distribution systems. Finally, it can be used to create off-grid renewable energy solutions that supply electricity to remote areas lacking access to conventional power networks.

Three US-based solar cell start-ups (Nanosolar, Nanosys, and Konarka Technologies) and corporate players, including Matsushita and STMicroelectronics, are striving to produce photon-harvesting materials at lower costs and in higher volumes than traditional crystalline silicon photovoltaic cells.^2^ Nanosys intends its solar coatings to be sprayed onto roofing tiles. And Konarka is developing plastic sheets embedded with titanium dioxide nanocrystals coated with light-absorbing dyes. The company acquired Siemens' organic photovoltaic research activities, and Konarka's recent $18 million third round of funding included the world's first- and fifth-largest energy companies, Electricité de France, and ChevronTexaco. If nanotech solar fabrics could be applied to, e.g., buildings and bridges, the energy landscape could change in significant ways. Integrated into the roof of a bus or truck, they could split water via electrolysis and generate hydrogen to run a fuel cell.

### 2.5 Sustainable cities and communities (Goal 11)

By using nano-coatings on windows to improve insulation and lower energy consumption, nanotechnology can be utilized to increase a building's energy efficiency [52] and decrease its environmental impact. Furthermore, cities can have access to clean, safe water thanks to nanotechnology-based water treatment technologies that increase the effectiveness of water treatment facilities and lessen the environmental impact of wastewater discharge. Air pollution [56,78] can be reduced in metropolitan areas by using air filters based on nanotechnology. Also, real-time infrastructure monitoring using nanotechnology-based sensors can help cities and populated areas be more resilient to natural catastrophes by seeing possible problems before they become serious. Lastly, urban transportation [33,[79], [80], [81], [82], [83]] networks can be improved with the use of nanotechnology (self-healing nano coatings, graphene for lighter and stronger vehicles' bodies, carbon-nanotubes bumper, and many others). Regarding Construction and buildings [[41], [42], [43],^45^], nanotechnology can create self-healing building materials, and improve insulation, coatings, and energy production. Concrete can patch gaps, resulting in more durable infrastructure and less maintenance. Nanoparticles can enhance thermal conductivity, increase energy efficiency, and enhance durability. Building-integrated photovoltaics (BIPV) can produce renewable energy on-site, reducing energy consumption in buildings. Mexico City faced significant air pollution issues. Nano-enabled coatings on buildings and roads, incorporating titanium dioxide nanoparticles [84], reduced air pollution by breaking down pollutants.^3^ This technology led to improved air quality, respiratory health, and a more sustainable urban environment, contributing to the goal of sustainable cities and communities.

### 2.6 Responsible consumption and production (Goal 12)

Nanomaterials can be employed to develop more durable and longer-lasting products [85], reducing waste generated by consumer goods. Furthermore, nanomaterials will help in developing more efficient recycling [44,45,55] processes, improving the sustainability of waste [53,55,86] management. Nanotechnology can make manufacturing processes more energy efficient, reducing energy consumption and greenhouse gas emissions. In addition, nanotechnology-based agricultural [[87], [88], [89], [90]] technologies can improve crop yields [[91], [92], [93], [94]] and reduce environmental impacts, promoting sustainable agriculture practices. Furthermore, nanotechnology-based water treatment technologies can improve the efficiency of water treatment plants and reduce the environmental impact of wastewater discharge.

### 2.7 Climate action (Goal 13)

Tackling climate change and environmental challenges is a critical issue today; setting ambitious goals, such as zero pollution approaches for a toxin-free environment, is a big challenge by itself. Major investment in cutting-edge technology and research has been considered where Nano plays a critical role in achieving these transformative obviatives. By using nanotechnology, renewable energy sources (Goal 7), like solar cells and fuel cells, can be made more effective and affordable, and versions of energy storage devices like batteries and capacitors can be made stronger and more effective. Furthermore, carbon dioxide emissions [38,95,96] from industrial operations can be captured and stored using nano-based materials, which lowers greenhouse gas emissions [[97], [98], [99]], using Nano-Zeolite, which can be achieved by increasing the energy efficiency of structures, transportation, and manufacturing techniques. Additionally, nanosensors can be used to continuously monitor environmental [53,56,60,83,[100], [101], [102]] conditions, spotting potential problems before they become serious and assisting in reducing the effects of climate change on ecosystems.

Tesla, a leader in electric vehicle manufacturing, utilizes nanotechnology in its battery technology. By using silicon nanowires in lithium-ion batteries [103], Tesla increased energy storage capacity and extended the driving range of electric vehicles. This innovation not only promotes clean energy but also reduces greenhouse gas emissions, supporting climate action.

Scientists at the Lawrence Berkeley National Laboratory, a part of the U.S. Department of Energy, have crafted an advanced carbon capture membrane with exceptional permeability. This innovation holds the potential to enhance the efficiency of isolating carbon dioxide from power plant emissions. By preventing the release of this greenhouse gas into the atmosphere, the membrane offers a promising avenue for mitigating climate change. The researchers concentrated their efforts on developing a hybrid membrane comprising both polymer and metal-organic framework components. The latter is a porous three-dimensional crystal renowned for its extensive internal surface area, capable of absorbing significant quantities of molecules.

Concrete, renowned for its strength and cost-effectiveness, stands as the ideal construction material. However, its durability diminishes over time, and substantial material is typically required to bear significant loads.

Introducing a small quantity of graphene (usually less than 1 %, often just 0.1 % of the entire mix) into the cement or overall concrete mixture yields various longevity benefits. These include a 25 % increase in tensile strength, a 35 % boost in compressive strength, and a four-fold improvement in water resistance. These enhanced physical properties also translate to a reduced requirement for material to support larger loads. Structures crafted with graphene-enhanced concrete have, to date, necessitated 33 % less material while maintaining comparable structural properties and durability.

By integrating graphene to reduce the volume of required material, we have the potential to slash concrete colossal 8 % global carbon footprint by up to 2 %. Several companies are already manufacturing graphene-enhanced cement, graphene-infused concrete mixtures, or graphene admixtures for existing concrete products. Key players, either with products on the market or in the prototype stage, include Global Graphene Group (GGG), Bio Graphene Solutions (BGS), GtM Action, C&O Concrete, First Graphene Limited, Haydale, Graphenea, and Black Swan Graphene.

Scientists at the CSIR-Indian Institute of Petroleum and Lille University of Science and Technology in France have made notable strides. They have successfully crafted a nanoCO_2_ harvester utilizing water and sunlight to convert atmospheric CO_2_ into methanol [104]. With applications as an engine fuel, solvent, antifreeze agent, and ethanol diluent, methanol is produced by enveloping spheres of copper zinc oxide and magnetite with a layer of modified graphene oxide. Resembling a miniature golf ball, this material surpasses traditional catalysts in CO_2_ capture efficiency and boasts reusability.

Meanwhile, a team led by Pulickel Ajayan at Rice University in Houston, Texas, has introduced a reusable nano-sponge designed to extract oil from contaminated seawater. Acknowledged for its efficacy in addressing oil spills, this material shows promise, although further scaling up is required for large-scale applications.

Cranfield University in the UK is currently working on six different projects for Climate change.

• Lambda Energy aims to increase crop yields using greenhouses coated with sunlight-changing films.
• SAGES London is exploring how natural, food-waste-derived dyes can be refined to produce more vibrant dyes and have commercial uses in other industries.
• Bluemethane is a project focused on optimizing technology to capture methane from water and create a new source of bioenergy.
• ManholeMetrics is developing low-cost long-life sensors that can be mounted to the underside of manhole covers to provide real-time flood warnings and enable flood prediction.
• TaiSan Motors Ltd: The next-generation battery pack for electric vehicles (EVs).
• Nium uses patent-pending nanotechnology to make green ammonia without direct CO_2_ emissions.

A recent trial in the UAE [105], featuring innovative cloud seeding technology, has yielded encouraging outcomes. The National Centre of Meteorology collaborated with the local weather association in Texas, conducting a series of flights to assess the effectiveness of the new approach. Nanomaterials took center stage in this endeavor. Designed explicitly for cloud seeding, these nanomaterials prove superior to conventional agents like salt and dry ice. Their precision and efficacy make them a more advanced tool for inducing rainfall from existing clouds.

### 2.8 Life below water (Goal 14)

In order to conserve and utilize ocean, sea, and marine resources sustainably, nanotechnology can help water bodies be effectively cleaned of pollutants [60], like heavy metals and organic compounds, by using nanoparticles. Additionally, nanotechnology-based sensors can accurately detect and monitor water pollution levels. In terms of oil spill cleanup [[106], [107], [108], [109]], nanotechnology provides innovative solutions. Nanostructured materials like graphene-based sorbents or nanofibers can effectively absorb oil from water surfaces due to their high surface area-to-volume ratios. Desalination [[61], [62], [63], [64]] is another area where nanotechnology plays a crucial role. Nanomaterials such as graphene oxide membranes or carbon nanotubes can selectively filter out salt ions from seawater, making it suitable for drinking and irrigation. Nanotechnology also contributes to marine biotechnology by enhancing drug delivery [110] systems for marine organisms. Nanoparticles can be designed to deliver therapeutic agents precisely to target cells or tissues in marine organisms, aiding in disease treatment and conservation efforts. To protect coral reefs [111], nanotechnology-based coatings can prevent the settlement of harmful organisms like algae or bacteria on their surfaces. These coatings [112] can also provide a controlled release of nutrients to promote coral growth. Nanosensors and nanodevices enable real-time monitoring of marine ecosystems by detecting changes in water quality, temperature, pH levels, and the presence of harmful substances. This data helps in the early detection of environmental issues and facilitates timely interventions. In terms of sustainable fishing practices [113], nanotechnology offers the development of nanoscale sensors to monitor fish populations, track migration patterns, and assess the impact of fishing activities on marine ecosystems. This information guides policymakers in implementing effective fisheries management strategies.

### 2.9 Life on land (Goal 15)

Nanomaterials can be used to remediate contaminated soils [56,114,115], improving soil quality and promoting plant growth. Furthermore, nanosensors can be used to monitor soil moisture levels, nutrient content, and other environmental factors in real time, improving the efficiency and sustainability of agricultural practices. Nanotechnology-based sensors can be used to monitor forest health and detect potential issues before they become critical, improving forest management practices and preventing deforestation [101]. In addition, nanotechnology-based materials can be used to develop new conservation technologies that protect endangered species and promote biodiversity [116]. It is also good to mention that nanotechnology-based materials can be used to restore degraded lands by improving soil quality and promoting plant growth.

The Fontagro^4^ project seeks to use nanotechnology (nanoclays and hydrogels) to help improve the moisture retention capacity of soils, increasing agricultural productivity in areas undergoing desertification and/or drought. Both materials will be developed in Ecuador by scientists from Yachay Tech University (UYT), evaluated under controlled conditions by INIAP (Ecuador), evaluated and validated in a participatory manner in real field conditions in four crops (potato, quinoa, corn, and wheat) by PROINPA (Bolivia) and by INIAP. The project strategy is based on developing and using nanoclays and hydrogels. Nanoclays have very small particles with a large contact and expansion surface. Hydrogels are manufactured from cellulose fibers obtained from agricultural waste and are inexpensive. Both materials have the ability to retain large volumes of water and have a low environmental impact, ideal for agricultural applications. After being developed in the laboratory, these products will be incorporated into degraded soils to see their effect on water retention, increasing microbial diversity, and improving agricultural productivity.

In Thailand, scientists at Chiang Mai University's nuclear physics laboratory have rearranged the DNA of rice by drilling a nano-sized hole through the rice cell's wall and membrane and inserting a nitrogen atom. So far, they've been able to change the color of the grain from purple to green. 5

Monsanto, Syngenta, and BASF are developing pesticides enclosed in nanocapsules or made up of nanoparticles. The pesticides can be more easily taken up by plants if they're in nanoparticle form; they can also be programmed to be “time released.” [117].

With funding from the US Department of Agriculture (10.13039/100000199USDA), 10.13039/100006498Clemson University researchers are feeding chickens bioactive polystyrene nanoparticles that bind with bacteria as an alternative to chemical antibiotics in industrial chicken production.

“Little Brother”: The USDA is pursuing a project to cover farmers’ fields and herds with small wireless sensors to replace farm labor and expertise with a ubiquitous surveillance system.

Once the **above-mentioned goals** have been achieved, this would pave the way to the following goals.

### 2.10 Zero hunger (Goal 2)

Sustainability is an essential driver for the agriculture and food sector, as it is crucial to feed all population with optimized health and nutritious produced food. Today's food sector can benefit significantly from Nanotechnology by achieving food security, improving nutrition, and promoting sustainable agriculture. By using nanoparticles to distribute nutrients [116,[118], [119], [120]] directly to plant roots and nanosensors to detect nutrient deficits in soil, new agricultural technologies [[87], [88], [89], [90],[121], [122], [123], [124]] can be created to boost crop yields and decrease waste (Goal 15). Furthermore, by incorporating silver, titanium dioxide, silver, gold, copper, and cadmium sulfide nanoparticles into packaging materials to provide a barrier against oxygen and moisture, nanotechnology can be used to build novel food packaging [[125], [126], [127], [128], [129]] materials that prolong food's shelf life and lower spoilage. Also, by using nanosensors to continuously monitor soil moisture levels, new water management technologies can be created that increase irrigation effectiveness and decrease water waste.

In Kenya, nanotechnology has been applied to improve soil quality. Nano-encapsulated fertilizer, slowly releasing nutrients to crops, enhanced crop yields by up to 30 %. This increased food production contributed to food security and improved income for local farmers, addressing both hunger and poverty.

Scientists from the University of Wisconsin have successfully used single bacterial cells to make tiny bio-electronic circuits, which could, in the future, be used to detect bacteria, toxins, and proteins^6^the medical nanotechnology market is anticipated to achieve a value.

Kraft, Nestlé, Unilever, and others are employing nanotech to change the structure of food – creating “interactive” drinks containing nanocapsules that can change color and flavor (Kraft) and spreads and ice creams with nanoparticle emulsions (Unilever, Nestlé) to improve texture. Others are inventing small nanocapsules that will smuggle nutrients and flavors into the body (what one company calls “nanoceuticals”).

BASF, Kraft, and others are developing new nanomaterials that extend food shelf life and signal when food spoils by changing color.

### 2.11 Good health and well-being (Goal 3)

Nanotechnology has the potential to contribute significantly to ensuring healthy lifestyles and promoting well-being for all people of all ages. It has the potential to enhance drug delivery, diagnostics, medical imaging, medical devices, and tissue engineering. Drug delivery devices [[110], [130], [131], [132], [133], [134], [135], [136]] on a nanoscale, using metallic and non-metallic nanoparticles and nanowires, can be designed to deliver medications directly to cancer cells, limiting damage to healthy cells and boosting therapeutic efficacy. Nanotechnology can also be used to create more sensitive and accurate diagnostic [[131], [135], [136], [137], [138], [139]] equipment, such as Nanosensors that identify diseases early on. Furthermore, nanoparticles can be utilized as contrast agents in Magnetic Resonance Imaging (MRI) images [131,135,137] to improve diagnosis accuracy (such as gold nanoshells, superparamagnetic Iron oxide nanoparticles coated with dextran, bismuth sulfide nanoparticles, gold nanoparticles, and quantum dots). Nanotechnology can also be used to create new medical devices [140] that are more effective and need fewer intrusive procedures than present approaches. Nanorobots, for example, can be utilized for targeted medicine administration or minimally invasive surgery. Nanotechnology can also be used in tissue engineering [132,[141], [142], [143], [144], [145], [146], [147]] to develop antioxidant and enhanced flexible nano-porous scaffolds that imitate the structure of human tissues and organs. These scaffolds [142,145,146,148] can subsequently be seeded with cells to form functioning tissues or organs suitable for transplantation. Nanoparticles can also be employed to incite cell growth and regeneration in tissues that have been injured. FDA (Food and Drug Administration) has also approved some nanomedicines such as Eligard (for Prostate cancer), Myocet (for breast cancer), Nanoxel (for Ovarian cancer, head, and neck cancer), and many others [131].

### 2.12 Quality education (Goal 4)

Ensuring inclusive and equitable quality education, as well as promoting lifelong learning opportunities for all, represents another vital challenge facing the world today – a challenge addressed by UNESCO Education Vision 2030.

Nanotechnology can be used to develop new educational environments, materials, and infrastructure that can enhance learning, such as nanoscale models of molecules and cells for teaching biology and chemistry. It can also be used to develop new energy-efficient lighting technologies that reduce energy consumption and improve the learning environment, such as using nanoscale LEDs [[149], [150], [151], [152]] to create brighter classroom lighting. Furthermore, nanotechnology can be used to develop new water purification [54,58,59] technologies that are more efficient and effective than current methods, ensuring that students have access to clean drinking water, as well as healthy buildings using nano-based strong concrete, isolating materials (aerogel) and coatings nanomaterials. It can also be integrated into new medical training technologies, providing students a more realistic and hands-on experience. Nanotechnology can also be used to promote science, Technology, Engineering, and mathematics (STEM) education [[153], [154], [155], [156], [157], [158]] by providing interactive and engaging learning experiences for students using nanoscale models and simulations.

### 2.13 Gender equality (Goal 5)

Gender equality is a fundamental human right and a necessary condition for sustainable development. It encompasses equal access to resources, opportunities, and decision-making power for people of all genders. While progress has been made in recent years, there is still a long way to go in achieving true gender equality. Nanotechnology, with its unique capabilities and applications, can impact this goal. One area where nanotechnology can contribute to gender equality is healthcare. Nanomedicine [138,143,159,160], the application of nanotechnology in medicine, has the potential to revolutionize healthcare by providing targeted drug delivery systems, disease early detection methods, and personalized treatments. This can have a significant impact on women's health, as many diseases affect women differently than men. For example, breast cancer [135,161] is more prevalent among women, and nanotechnology-based diagnostic tools can enable early detection and improve treatment outcomes.

Furthermore, nanotechnology can also address menstrual health and hygiene challenges [162]. In many parts of the world, lack of access to affordable and hygienic [163,164] menstrual products hinders young girls' education and women's participation in society. Nanofiber-based materials can be used to develop highly absorbent and biodegradable sanitary pads [165] that are more comfortable and environmentally friendly than traditional options. Additionally, nanotechnology can be employed to create antimicrobial coatings for reusable menstrual cups, reducing the risk of infections. Another area where nanotechnology can contribute to gender equality is agriculture. Women play a vital role in agricultural production, particularly in developing countries. However, they often face numerous challenges, such as limited access to resources, climate change [91] impacts, and post-harvest losses. Nanotechnology can address these challenges by improving crop yields [[91], [92], [93], [94]], enhancing soil fertility with nano-fertilizer (NH^4+^-N), which recorded lower N_2_O emission (1.8 mg m^−2^day^−1^) than conventional fertilizer (2.7 mg m^−2^day^−1^) as well as using Zinc Oxide, Silicon dioxide, Titanium dioxide, and many others, as well as developing efficient pest control [[166], [167], [168], [169]] methods using metal oxide nanomaterials.

Furthermore, nanosensors can monitor soil moisture and nutrient levels, enabling precise irrigation and fertilizer application. This can empower women farmers by increasing their productivity and income. In the energy field, nanotechnology can contribute to gender equality by promoting access to clean and affordable energy sources. In many developing countries, women and girls are burdened with collecting firewood or using inefficient cooking methods, which negatively impacts their health and well-being. Nanotechnology-based solutions such as solar cells, energy storage devices, and efficient lighting systems can provide sustainable energy alternatives. By reducing the time spent on energy-related chores, women and girls can have more opportunities for education, economic empowerment, and personal development.

### 2.14 Reduced inequalities (Goal 10)

Reducing inequalities is a crucial goal for sustainable development, as it aims to ensure that everyone has equal access to resources, opportunities, and essential services. Nanotechnology, with its unique properties and applications, has the potential to contribute significantly to reducing Inequalities. One area where nanotechnology can make a difference is in healthcare [163,164]. Access to quality healthcare is often limited in remote or underserved areas, leading to health disparities and inequalities. Nanotechnology-based medical devices and diagnostic tools can help bridge this gap by providing affordable and portable solutions. For example, nanosensors can be used for rapid and accurate disease detection, enabling early intervention, and reducing healthcare disparities. Nanoparticles can also be utilized for targeted drug delivery, increasing the effectiveness of treatments while minimizing side effects.

Furthermore, nanotechnology can contribute to reducing inequalities in access to clean water [54,56,58] and sanitation. In many parts of the world, lack of access to safe drinking water and proper sanitation facilities leads to health problems and perpetuates poverty. Nanomaterials can be employed to develop efficient water purification systems that remove contaminants and pathogens effectively. Nanostructured membranes can enhance water filtration processes, making clean drinking water more accessible in resource-limited settings. Additionally, nanotechnology can enable the development of self-cleaning surfaces [9,170] and antimicrobial [164,171] coatings for sanitation facilities using nano ZnO and carbon nanotubes, improving hygiene standards. Education [153,154,156] is another area where nanotechnology can help reduce inequalities. Access to quality education is often limited in disadvantaged communities due to various factors, such as lack of resources or qualified teachers. Nanotechnology-based educational tools and materials can provide interactive learning experiences that are engaging and accessible to all students. For instance, nanoscale models or simulations can be used to visualize complex scientific concepts, making them easier to understand. Nanotechnology can also contribute to developing low-cost educational devices and platforms, enabling remote learning opportunities for underserved populations. In the energy field [29,30,52,65,66], nanotechnology can help reduce inequalities by promoting access to clean and affordable energy sources. Many developing regions still rely on fossil fuels or inefficient energy systems, which disproportionately affect marginalized communities. Nanotechnology-based solutions such as solar cells, energy storage devices, and energy-efficient materials can provide sustainable alternatives. By decentralizing energy production and reducing reliance on centralized grids, nanotechnology can empower communities to generate their own clean energy and reduce energy poverty.

### 2.15 No poverty (Goal 1)

By developing new, more affordable, green, and effective materials and devices than those produced by present technologies to be applied in construction and building in energy conversion and energy storage, in health, water treatment, and agriculture, nanotechnology can aid in the fight against poverty. Thus, by transferring these technologies to those countries facing high levels of poverty, identifying, and using their existing raw materials, establishing factories, and creating new job opportunities, the poverty level can be decreased.

In India, poverty is intertwined with inadequate access to clean drinking water. Nanotechnology was employed to develop low-cost water filters with silver nanoparticles. These filters effectively remove bacteria and impurities, providing safe drinking water. As a result, impoverished communities experienced reduced waterborne diseases, improved health, and lower medical expenses, contributing to poverty reduction.

The University of Toronto Joint Center for Bioethics (UTJCB) has consistently engaged the academic community by presenting a map illustrating various governmental nanotechnology initiatives. This map underscores the inclination of numerous developing countries to promote these technologies. Highlighting the potential of several nanotechnologies to ameliorate impoverished living conditions, it is observed that developing countries are actively pursuing nanotech initiatives. The UTJCB suggests the necessity of establishing an international network to evaluate emerging technologies geared toward development. China, South Korea, and India are recognized as leaders, while Thailand, the Philippines, South Africa, Brazil, and Chile fall within the intermediate category. Argentina and Mexico are identified as emerging participants. According to the authors, governments' willingness to foster nanotechnologies serves as an indicator of the perceived value of these technologies as instruments for development. The potential for more affordable and widely available solar energy, novel methods for water remediation, and swift, cost-effective disease diagnosis is a justification for the relevance of nanotechnologies to underserved populations in developing countries. In the developing world, nanotechnology is anticipated to play a pivotal role due to its minimal labor, land, and maintenance requirements. Recognized for its high productivity, cost-effectiveness, and modest material and energy demands, nanotechnology products are expected to be remarkably efficient as energy producers, materials collectors, and manufacturing equipment [172,173].

### 2.16 Decent work and economic growth (Goal 8)

In the past two decades, interest in nanotechnology, a relatively young field of science, has grown, and it is now quickly moving from the academic world into the business world. It has been predicted that nanotechnology will have more than three trillion-dollar influence on the world economy due to the potential breakthroughs it may bring about [174]. Nanotechnology has the potential to drive economic growth and create new job opportunities in various sectors, including research and development, manufacturing, energy, healthcare, and agriculture. The development of nanotechnology requires significant research and development, which can create jobs for scientists, engineers, and technicians [33,[79], [80], [81],^85^,^127^]. Manufacturing nanotechnology-based products requires specialized processes and can create jobs in the manufacturing sector. Nanotechnology can also improve energy efficiency and develop renewable energy sources, creating new job opportunities in the energy sector. Additionally, nanotechnology-based medical devices and therapies can improve healthcare outcomes and create jobs in the healthcare industry. Lastly, nanotechnology can be used to develop new agricultural technologies that will enhance crop yields, creating job opportunities in the agriculture sector.

Establishing companies is a crucial gauge for assessing a novel technology's advancement and economic significance. Typically, these newly formed companies are startups possessing a primary asset: a patent on an innovative technology that they can either exploit independently or license to other companies with more outstanding capabilities in terms of production or distribution. In this high-tech and, consequently, high-risk sector, Venture Capital emerges as a significant source of financing.

Regarding job creation, startups and small to medium-sized enterprises (SMEs) play a pivotal role. While many employment opportunities will arise in SMEs, established companies have also embraced nanotechnology, in recent years, to sustain their competitiveness. This explains the identification of companies as nanotech-oriented, some of which have existed for over a century. Noteworthy examples include major companies in the chemical and pharmaceutical industry, optics, and electronics (such as Bayer, BASF, Carl Zeiss, Agfa-Gevaert, General Electric, and Philips, all established before 1900). However, it is crucial to note that these well-established companies constitute a minority among all existing nanotech companies.

By 2026, 7 the medical nanotechnology market is anticipated to achieve a value of $461,252 million, with an estimated Compound Annual Growth Rate (CAGR) of around 11.9 % between 2021 and 2026. The convergence of nanotechnology and medicine opens up exciting prospects, holding the potential to combat some of the most challenging diseases humanity faces, such as Parkinson's, Alzheimer's, and diabetes.

According to nanotechnology industry revenue statistics, the nanomaterials market is poised to reach $98.9 billion by 2025, with a projected CAGR of 13.9 %. A significant portion of this market value is expected to originate from Japan, which will contribute $6.9 billion by 2025.

The global nanotechnology market is forecasted to reach $306.1 billion by 2025, marking a substantial increase from its 2018 value of $160.8 billion. Experts predict a Compound Annual Growth Rate (CAGR) of 9.6 % from 2018 to 2025.

Nanoparticles constitute 85 % of the nanotech market, showcasing nanotechnology's diverse applications and far-reaching impacts on society.

In a significant move, the 2021 budget plan of the U.S. President allocated over $1.7 billion for the National Nanotechnology Initiative. Given the remarkable advancements in nanotechnology, there is a compelling case for increased investment across various sectors, from medicine to environmental technology, as the potential seems boundless.

**The Achievement of all the previous goals will promote peaceful and inclusive societies for sustainable development, provide access to justice for all, and build** effective, accountable, and inclusive institutions at all levels.

### 2.17 Peace, justice, and strong institutions (Goal 16)

Nanotechnology, with its unique capabilities and applications, can play a significant role in advancing peace [175]. Forensic investigations are crucial in ensuring justice and maintaining law and order. Nanotechnology-based techniques can enhance forensic analysis [[176], [177], [178]] by providing more accurate and efficient evidence collection and analysis methods. For example, nanosensors can detect trace amounts of substances such as drugs or explosives, enabling faster and more precise identification. Nanoparticles can also be employed as contrast agents in imaging techniques, improving the visualization of evidence such as fingerprints or DNA samples.

Furthermore, nanotechnology can aid in strengthening institutions through improved security measures [[179], [180], [181]]. Nanomaterials can be utilized to develop advanced security systems with enhanced threat detection and prevention capabilities. For instance, nanoscale sensors integrated into surveillance systems can detect abnormal activities or hazardous substances in real time, enhancing public safety. Nanotechnology-based encryption methods can also improve data security and protect sensitive information, contributing to the establishment of robust institutional frameworks, especially in the era of quantum computing. Nano is realizing stable qubits, thus leading to easy and rapid encryption and decryption of information and data analysis. Nanotechnology can also contribute to promoting peace by addressing environmental challenges [101]. Environmental degradation often leads to conflicts over resources such as water or land. Nanotechnology-based solutions can help mitigate these conflicts by providing sustainable alternatives. For example, nanomaterials can be used for efficient water purification or wastewater treatment, reducing the strain on freshwater resources. Nanotechnology-enabled sensors can monitor air quality, soil health, and water pollution, enabling early detection and prevention of environmental hazards. By promoting sustainable resource management, nanotechnology can contribute to peacebuilding efforts.

Additionally, nanotechnology can support access to justice by improving legal processes and enhancing forensic capabilities. Nanoscale materials can be used to develop advanced materials for evidence preservation, ensuring the integrity of collected samples. Nanotechnology-based techniques can also improve the analysis of complex legal documents or contracts, facilitating more efficient and accurate legal decision-making. Furthermore, nanotechnology can contribute to developing secure and tamper-proof identification systems, ensuring the authenticity of legal documents, and preventing identity theft or fraud. The Internet of Nanothings (IoNT) [[181], [182], [183]] is an emerging field that combines nanotechnology and the Internet of Things (IoT). It involves the integration of nanoscale devices, sensors, and systems into the IoT framework, enabling communication and data exchange at the nanoscale level. As nanodevices collect and transmit sensitive data, ensuring data integrity, confidentiality, and protection against cyber-attacks becomes crucial.

It is worth mentioning that during war and conflict, for early warning and threat detection, nanosensors [[184], [185], [186]] can detect chemical agents, explosives, biological threats, and other hazardous substances more accurately and rapidly than traditional sensors, enabling rapid response measures to protect people from potential dangers.

Nanocoating materials can effectively absorb or scatter radar waves [48,187,188], making items undetectable by radar and, thus, improving stealth capabilities (such as Ghost ships, and ghost airplanes).

By incorporating nanocapsules with healing agents, self-healing materials [20,79,189] can be fabricated and used to repair damage caused by impacts or wear and tear automatically.

During war and conflict, people face challenges adapting to these extreme environments. Nanofabricated clothing [[190], [191], [192]] can provide better insulation or cooling properties in extreme temperatures or protect against chemical or biological agents.

It is good to mention that researchers are now developing non-lethal weapons [193] that incapacitate or immobilize enemies without causing significant harm. This approach aims to minimize casualties and reduce the overall intensity of conflicts, potentially leading to more peaceful outcomes.

“We knew the world would not be the same. A few people laughed; a few people cried. Most people were silent. … … ‘Now I am become Death, the destroyer of worlds.’ I suppose we all thought that one way or another.” said Robert Oppenheimer in 1965. It is important to note that while nanotechnology can contribute to peacebuilding efforts during the war, its effectiveness ultimately depends on the intentions and actions of those involved in the conflict. Ethical considerations and responsible deployment of nanotechnology are crucial to ensure its positive impact on establishing peace.

## 3 Moving Forward and future directions

Scientists must recognize that nanotechnology benefits (from economic, social, and environmental perspectives) can only be achieved once these findings are commercialized [194] and deployed at a large scale, taking into consideration the intellectual property protection, the target market, the collaborators, and enough funds.

Scientists are keen on achieving positive impacts. They proactively understand society's needs and potential environmental impacts, considering the social acceptability, stakeholders' concerns, and commercial aspects.

To accomplish this, all stakeholders must collaborate and work together to promote nanotechnology research and potential applications (through awareness campaigns), promote education and training at school levels, encourage industries to collaborate with universities on nanotechnology applied research related to their needs considering it as part of their corporate social responsibility projects (CSR), as well as government support to green nanotechnology research, commercialization, and industrialization.

On the other hand, scientists are still investigating the advancement of environmentally friendly and sustainable nanomaterials that limit environmental harm and decrease reliance on scarce components. Scientists are researching alternative materials that are lead-free and do not depend on precious resources, including rare earth elements. Their goal is to encourage sustainable manufacturing processes and improve the eco-efficiency of nanotechnology applications in many industries. These endeavors aid in achieving the ultimate objective of sustainable development by reducing environmental harm and enhancing the efficient use of resources over the lifecycle of nano products. By continuously conducting research and working together, incorporating environmentally friendly nanomaterials shows potential for promoting sustainable development objectives while reducing negative environmental impacts.

Thus, the potential future directions and trends of nanotechnology [[195], [196], [197], [198], [199], [200], [201], [202], [203], [204]] about the 17 Sustainable Development Goals (SDGs) are extensive and varied and, based on all the above, can be summarized in different domains to be further investigated.

1. Healthcare (SDG 3: Good Health and Well-being) [201,203,204]: Progress in developing nanoparticle-based targeted drug delivery systems to enhance the accuracy and effectiveness of illness treatment. The aim is to create nanoscale diagnostic instruments that can identify diseases at an early stage, hence improving the accessibility and effectiveness of healthcare. The fusion of nanotechnology with regenerative medicine will facilitate tissue engineering and the regeneration of organs.
2. SDG 6, Clean Water and Sanitation, focuses on improving access to clean water and sanitation. One way to do this is by using nanomaterials in water purification technologies, such as nanofiltration membranes and nano adsorbents, to boost the effectiveness of the purification process. The objective is to create nanosensors that continuously monitor water quality and detect pollutants in real-time. This will allow for proactive management of water resources.
3. The focus is on Renewable Energy (SDG 7: Affordable and Clean Energy). Specifically, the research involves investigating nanomaterials [195,197,198,205,206] to develop more efficient energy storage options, including high-capacity batteries and supercapacitors. The objective is to incorporate nanotechnology into photovoltaic systems to improve solar cells' efficiency and decrease the expenses associated with their production. The aim is to create nanocatalysts to enhance the efficiency of converting renewable energy sources, such as hydrogen and biofuels.
4. Sustainable Agriculture (SDG 2: Zero Hunger): Nanotechnology is used in precision agriculture to distribute nutrients and agrochemicals [196,198,199,201] directly to specific areas, minimizing environmental harm. The objective is to create nanoscale sensors to monitor soil health, crop development, and insect infestations. These sensors will help farmers improve their agricultural operations. The use of nanomaterials in the development of controlled-release formulations for fertilizers and pesticides aims to enhance resource efficiency and increase agricultural yields.
5. Environmental Remediation (SDG13: Climate action and Multiple SDGs): The utilization of nanomaterials [195,197,[201], [202], [203]] to mitigate and rectify pollution, encompassing the elimination of heavy metals, organic pollutants, and harmful compounds from the atmosphere, land, and water. The objective is to create nanoscale filtration systems that may be used for wastewater treatment. These systems will help ensure the safe reuse of water resources and minimize environmental contamination.
6. Infrastructure and Urban Development (SDG 11: Sustainable Cities and Communities): Incorporating nanotechnology into construction materials to improve the longevity, strength, and energy efficiency of buildings and infrastructure. The aim is to use nanoparticles to create coatings that possess the potential to self-heal and self-clean. This development will result in reduced maintenance expenses and enhanced urban livability. Using nanosensors for immediate monitoring of infrastructure integrity and environmental factors enhances the safety and sustainability of metropolitan areas.
7. Education and Capacity Building (SDG 4: Quality Education): Incorporating nanotechnology into the educational curriculum to promote STEM (Science, Technology, Engineering, and Mathematics) literacy and encourage innovative abilities. Implementing nanotechnology research and training initiatives aims to provide future generations with the necessary knowledge and skills to tackle worldwide issues effectively.

The probable future possibilities highlight nanotechnology's diverse role in promoting sustainable development in all 17 SDGs, providing creative solutions to intricate social and environmental problems. Continued exploration, cooperation, and financial support in nanotechnology are crucial for fully harnessing its potential in advancing the Sustainable Development Goals (SDGs).

## 4 Challenges and risks associated with the use of nanotechnology serving the 17 SDGs

Nanotechnology holds great promise in addressing the UN SDGs by offering innovative solutions across its various sectors. However, this emerging technology, like any other, poses particular challenges and risks associated with the use of nanomaterials at the nanoscale. These challenges and risks can be categorized as follows.

1. **Health and environmental concerns** [[207], [208], [209], [210], [211], [212], [213], [214], [215]], coming from releasing nanomaterials, which have unique properties, into the environment can pose human and ecological risks (such as toxicity and bioaccumulation) and potential harm to ecosystems. Researchers and policymakers should collaborate to develop guidelines for the safe use and disposal of nanomaterials, ensuring minimal environmental impact. Implementing strict safety protocols in workplaces and conducting thorough toxicity assessments are crucial. Developing safer nanomaterials and responsible disposal practices can also help mitigate these risks. By mitigating these risks, we contribute to SDG2 (No Hunger) and SDG 3 (Good Health and Well-being), ensuring healthy lives and promoting well-being for all ages. Furthermore, efforts to minimize environmental impact align with SDG 6 (Clean Water and Sanitation), SDG7, SDG11, SG13, SDG14, and SDG 15 (Life on Land), contributing to the conservation of ecosystems and resources.
2. **Regulatory challenges** [207,208,213], since the rapid pace of nanotechnology development outstrips regulatory processes and standards, potentially leading to unanticipated risks. Regulatory bodies should collaborate with scientists and industry to develop adaptive regulatory frameworks. Standardization efforts can also help ensure the safe and effective use of nanomaterials. By establishing effective regulations, we contribute to SDG 9 (Industry, Innovation, and Infrastructure), fostering sustainable industrialization and innovation. Moreover, proper regulation promotes responsible consumption and production, supporting SDG 12.
3. **Ethical and Social Implications** [[215], [216], [217]], related to privacy, security, equity, and the responsible use of powerful technologies. Open dialogue and public engagement are essential. Ethical guidelines and regulatory frameworks should be established to address societal concerns and ensure equitable access to nanotechnological advancements. By fostering inclusivity and addressing inequalities, we contribute to SDG 5 (Gender Equality) and SDG 10 (Reduced Inequality). Furthermore, promoting peaceful and inclusive societies through ethical governance aligns with SDG 16 (Peace, Justice, and Strong Institutions).
4. **Cost and Accessibility,** [[207], [208], [215]] Since nanotechnology offers cost-effective solutions based on the fact of using fewer materials with high and unique properties, the initial costs of research, development, and implementation can still be high. Ensuring the affordability and accessibility of nanotechnology-based solutions, especially in low-resource settings, is a significant challenge. By making innovative technologies accessible to all, we contribute to SDG 1 (No Poverty), SDG7 (Affordable and Clean Energy), and SDG8 (Decent Work and Economic Growth), fostering economic opportunities and eradicating poverty.
5. **Technology transfer and capacity building:** [207,210,215,218,219] Many countries may lack the infrastructure, expertise, and resources to harness the benefits of Nanotechnology. Encouraging responsible technology transfer and ensuring that nanotechnological innovations benefit all segments of society is critical. Promoting open access to research, fostering technology-sharing agreements, and creating mechanisms for fair licensing can help address these barriers and ensure the broader application of nanotechnology. Investments in education and workforce development can help people adapt to changing job markets, contributing to SDG 4 (Quality Education), SDG 8 (Decent Work and Economic Growth), and SDG 9 (Industry, Innovation, and Infrastructure). Moreover, fostering international cooperation and partnerships aligns with SDG 17 (Partnerships for the Goals), ensuring that nanotechnological innovations benefit all segments of society.
6. **Intellectual property and innovation,** [[207], [209], [213], [215], [219], [220]] since Intellectual property rights can hinder the dissemination of nanotechnology solutions, particularly in resource-constrained regions. Promoting open access to research, fostering technology-sharing agreements, creating mechanisms for fair licensing, and balancing the need for innovation with IP Protection can help address these barriers and ensure the broader application of nanotechnology. Thus, by fostering open access to research and fair licensing mechanisms, we contribute to SDG 9 (Industry, Innovation, and Infrastructure) and SDG 10 (Reduced Inequality). Ensuring fair access to nanotechnology innovations helps reduce inequalities and promotes inclusive development. Furthermore, it fosters international collaboration supporting SDG17(Partnership for the Goals). Furthermore, by ensuring fair access to nanotechnology innovations, we contribute to promoting innovation and sustainable development in urban areas, aligning with the goals of SDG 11.
7. **Public perception and acceptance** [207,213,215] of Nanotechnology can significantly influence its adoption and implementation. It is crucial to address public concerns, foster transparency, and promote public engagement to build trust and acceptance of nanotechnology-based solutions. That's why scientific illustrations of recent findings during the research phase are essential for communicating with the public. By promoting public trust and acceptance, we contribute to SDG 16 (Peace, Justice, and Strong Institutions) and SDG 17 (Partnerships for the Goals), fostering stakeholder collaboration and promoting inclusive development. Furthermore, we contribute to SDG 13 (climate change) and its efforts to combat climate change.

Table 4 summarizes the proposed link between the identified risks and challenges and the 17 SDGs.

**Table 4:** Link between the Challenges and Risks of using Nanotechnology and the 17 UN SDGs.

	Health and environmental concerns	Regulatory challenges	Ethical & Social Implications	Cost and Accessibility	Intellectual property and innovation	Technology transfer and capacity building	Public Perception and acceptance
Goal 1: No Poverty				X			
Goal 2: Zero Hunger	X						
Goal 3: Good Health and well-Being	X						
Goal 4: Quality Education						X	
Goal 5: Gender Equality			X				
Goal 6: Clean Water and Sanitation	X						
Goal 7: Affordable and Clean Energy	X			X			
Goal 8: Decent Work and Economic Growth				X		X	
Goal 9: Industry, Innovation, and Infrastructure		X			X	X	
Goal 10: Reduced Inequality			X		X		
Goal 11: Sustainable Cities and Communities	X				X	X	
Goal 12: Responsible Consumption and Production		X					
Goal 13: Climate Action	X						X
Goal 14: Life Below Water	X						
Goal 15: Life on Land	X						
Goal 16: Peace, Justice, and Strong Institutions			X				X
Goal 17: Partnerships for the Goals					X	X	X

Nanotechnology applications may have unforeseen consequences, such as unintended side effects or disruptions to existing systems. It is crucial to conduct comprehensive risk assessments and monitor the long-term impacts of nanotechnology interventions.

By recognizing and addressing these challenges, stakeholders can work together to maximize nanotechnology's benefits while minimizing potential risks. A holistic and responsible approach is essential to leveraging nanotechnology's potential to drive progress towards the SDGs safely and equitably.

## 5 Conclusion

As a general-purpose technology that can solve problems and challenges at different levels in society, nanotechnology holds immense promise in addressing the global challenges outlined by the United Nations Sustainable Development Goals (SDGs). By manipulating materials at the nanoscale, researchers have unlocked new possibilities in various fields, including energy, healthcare, agriculture, and environmental conservation, by exploring the new properties of materials at the atomic scale, increasing their efficiency, improving their properties, and minimizing their size. Thus, by manipulating the atoms and improving their properties, new areas can be explored and improved, serving our world's challenges. Moreover, nanotechnology can play a crucial role in sustainable agriculture by improving crop yields, developing efficient nutrient delivery systems, and implementing effective pest control mechanisms. Additionally, it offers innovative solutions for environmental remediation and pollution control. Responsible research and development practices must be upheld to ensure the safe and sustainable deployment of nanotechnology.

Collaboration across disciplines is crucial to maximize the positive impact of nanotechnology on sustainable development. Nanotechnology presents a transformative opportunity to address global challenges and advance sustainable development. We can make significant strides toward achieving the UN Sustainable Development Goals by leveraging its unique properties and processes. Moreover, the cross-cutting effects of nanotechnology enable a holistic approach to addressing these goals, creating synergies that amplify the positive impacts. However, responsible development, regulation, and equitable access to nanotechnology are crucial to minimize potential trade-offs and ensure that the benefits are realized for all, ultimately contributing to a more sustainable and equitable global future. The successful integration of nanotechnology into the SDGs represents a blueprint for harnessing cutting-edge science and technology for the betterment of humanity and the planet. It is imperative that we continue to explore and invest in this promising field to create a more sustainable future for all. However, as with any emerging technology, it is essential to consider the ethical implications and potential risks associated with nanotechnology applications.

## CRediT authorship contribution statement

**Basma Elzein:** Validation, Supervision, Resources, Project administration, Methodology, Investigation, Formal analysis, Conceptualization.

## Declaration of competing interest

The authors declare that they have no known competing financial interests or personal relationships that could have appeared to influence the work reported in this paper.

## Footnotes

1 https://www.pub.gov.sg/Public/WaterLoop/OurWaterStory/NEWater.

2 ”Forbes Category killers: 5 nanotechnologies that could change the world”, September 2004 http://www.forbesinc.com/newsletters/nanotech/public/samples/Nanotech_5technologies.pdf.

3 https://blogs.rochester.edu/thegreendandelion/2013/11/mexicos-smog-eating-building/.

4 https://www.fontagro.org/new/proyectos/nanotecnologiahumedales/en.

5 https://www.oecd.org/science/nanosafety/44108334.pdf.

6 Robert J. Hamers et al. ”Manipulation and Real-Time Electrical Detection of Individual Bacterial Cells at Electrode Junctions: A Model for Assembly of Nanoscale Biosystems” Nano Letters April 2005. First presented at American Chemical Society, March 2005 http://pubs.acs.org/journals/nalefd/.

7 https://seedscientific.com/nanotechnology-statistics/.

## References

[1] Feynman R.P. (1960). There's Plenty of Room at the Bottom. Eng. Sci..

[2] Taniguchi N. (1974).

[3] Binnig G., Rohrer H., Gerber C., Weibel E. (1982). Tunneling through a controllable Vacuum gap. Appl. Phys. Lett..

[4] (1991). Unbounding the future: the nanotechnology revolution. Precis. Eng..

[5] Eigler D.M., Schweizer E.K. (1990). Positioning single atoms with a Scanning Tunnelling microscope. Nature.

[6] Iijima S. (1991). Helical microtubules of graphitic carbon. Nature.

[7] Kroto H.W., Heath J.R., O'Brien S.C., Curl R.F., Smalley R.E. (1985). C60: buckminsterfullerene. Nature.

[8] Karthick B., Maheshwari R. (2008). Lotus-inspired nanotechnology applications. Resonance.

[9] Sas I., Gorga R.E., Joines J.A., Thoney K.A. (2012). Literature review on superhydrophobic self-cleaning surfaces produced by electrospinning. J. Polym. Sci., Part B: Polym. Phys..

[10] Qi M., Liu Y., Li T. (2013). Nano-TiO2 improve the photosynthesis of tomato leaves under mild heat stress. Biol. Trace Elem. Res..

[11] Bhattacharyya G., Oliveira P., Krishnaji S.T., Chen D., Hinman M., Bell B., Harris T.I., Ghazitabatabaei A., Lewis R.V., Jones J.A. (2021). Large scale production of synthetic spider silk proteins in Escherichia coli. Protein Expr. Purif..

[12] Poddar H., Breitling R., Takano E. (2020). Towards engineering and production of artificial spider silk using tools of synthetic biology. Engineering Biology.

[13] Gour A., Ramteke S., Jain N.K. (2021). Pharmaceutical applications of quantum dots. AAPS PharmSciTech.

[14] Zhang Y., Liu B., Liu Z., Li J. (2022). Research progress in the synthesis and biological application of quantum dots. New J. Chem..

[15] Khan I., Saeed K., Khan I. (2019). Nanoparticles: properties, applications and toxicities. Arab. J. Chem..

[16] Stark W.J., Stoessel P.R., Wohlleben W., Hafner A. (2015). Industrial applications of nanoparticles. Chem. Soc. Rev..

[17] Gonzalez-Pedro V., Zarazua I., Barea E.M., Fabregat-Santiago F., de la Rosa E., Mora-Sero I., Gimenez S. (2014). Panchromatic solar-to-H-2 conversion by a hybrid quantum dots-dye dual absorber tandem device. J. Phys. Chem. C.

[18] Etgar L., Moehl T., Gabriel S., Hickey S.G., Eychmuller A., Gratzel M. (2012). Light energy conversion by mesoscopic PbS quantum dots/TiO2 heterojunction solar cells. ACS Nano.

[19] Jackson T.C., Patani B.O., Ekpa D.E. (2017). Nanotechnology in diagnosis: a review. Adv Nanopart.

[20] Ghorbani M., Ebrahimnezhad-Khaljiri H., Eslami-Farsani R., Vafaeenezhad H. (2021). The synergic effect of microcapsules and titanium nanoparticles on the self-healing and self-lubricating epoxy coatings: a dual smart application. Surface. Interfac..

[21] ElZein B., Abulikemu M., Barham A.S., Al-Kilani A., Alkhatab M.I., Hamdan S.M., Dogheche E., Jabbour G.E. (2022). In situ growth of PbS nanoparticles without organic linker on ZnO nanostructures via successive ionic layer adsorption and reaction (SILAR). Coatings.

[22] Abdalla S., Al-Marzouki F., Al-Ghamdi A.A., Abdel-Daiem A. (2015). Different technical applications of carbon nanotubes. Nanoscale Res. Lett..

[23] Pang J., Bachmatiuk A., Yang F., Liu H., Zhou W., Rümmeli M.H., Cuniberti G. (2021). Applications of carbon nanotubes in the Internet of Things era. Nano-Micro Lett..

[24] Castro-Rojas M.A., Vega-Cantu Y.I., Cordell G.A., Rodriguez-Garcia A. (2021). Dental applications of carbon nanotubes. Molecules.

[25] Saliev T. (2019). The advances in biomedical applications of carbon nanotubes. C (Basel).

[26] Ha H., Amicucci C., Matteini P., Hwang B. (2022). Mini review of synthesis strategies of silver nanowires and their applications. Colloids and Interface Science Communications.

[27] Jiu J., Suganuma K. (2016). Metallic nanowires and their application. IEEE Trans Compon Packaging Manuf Technol.

[28] Zhang Y., Ram M.K., Stefanakos E.K., Goswami D.Y. (2012). Synthesis, characterization, and applications of ZnO nanowires. J. Nanomater..

[29] El Zein B., Dogheche E. (2013).

[30] Leprince-Wang Y., Jing G., El Zein B. (2023). Novel ZnO-based nanostructures: synthesis, characterization and applications. *Crystals*. MDPI February.

[31] Kerkache L., Layadi A., Hadjersi F., Dogheche E., Gokarna A., Stolz A., Halbwax M., Vilcot J.P., Decoster D., El Zein B., Habib S.S. (2010). Sputtered indium tin oxide thin films deposited on glass substrate for photovoltaic application. Renewable Energy and Power Quality Journal.

[32] Yakoub S.E., Kashyout A.E.-H.B., Shoueir K., El-Kemary M. (2023). Design and performance analyses of graphene-nano plasmonic devices for wireless gas sensor applications. Int. J. Hydrogen Energy.

[33] Ekengwu I.E., Utu O.G., Okafor C.E. (2019). Nanotechnology in automotive industry: the potential of graphene. IRE Journals.

[34] Moon E., Kim J., Nam S., Eom S. (2012). The application of graphene as a support for cathode materials of metal-air batteries. Jpn. J. Appl. Phys..

[35] Wang J.T.W., Ball J.M., Barea E.M., Abate A., Alexander-Webber J.A., Huang J., Saliba M., Mora-Sero I., Bisquert J., Snaith H.J., Nicholas R.J. (2014). Low-temperature processed Electron collection layers of graphene/TiO2 nanocomposites in thin film perovskite solar cells. Nano Lett..

[36] Gertman R., Osherov A., Golan Y., Visoly-Fisher I. (2014). Chemical bath deposited PbS thin films on ZnO nanowires for photovoltaic applications. Thin Solid Films.

[37] El Zein B., Boulfrad S., Jabbour G.E., Dogheche E. (2014). Parametric study of self-forming ZnO nanowall network with honeycomb structure by pulsed laser deposition. Appl. Surf. Sci..

[38] Elrashidi A., Traversa E., Elzein B. (2022). Highly sensitive ultra-thin optical CO2 gas sensors using nanowall honeycomb structure and plasmonic nanoparticles. Front. Energy Res..

[39] El Zein B., Boulfrad S., Jabbour G.E., Dogheche E. (2014). Parametric study of self-forming ZnO nanowall network with honeycomb structure by pulsed laser deposition. Appl. Surf. Sci..

[40] Aithal S.P.S.A. (2021).

[41] Jia Z.-M., Zhao Y.-R., Shi J.-N. (2023). Adsorption kinetics of the photocatalytic reaction of nano-TiO2 cement-based materials: a review. Construct. Build. Mater..

[42] He W., Li S., Jiao Z., Wang N., Xu J., Zhou J., Zhao Q. (2023). Effect of regenerated nano-FeB on mechanical properties of cement paste. Construct. Build. Mater..

[43] He S., Chai J., Yang Y., Cao J., Qin Y., Xu Z. (2023). Effect of nano-reinforcing phase on the early hydration of cement paste: a review. Construct. Build. Mater..

[44] Rezaei F., Memarzadeh A., Davoodi M.-R., Dashab M.-A., Nematzadeh M. (2023). Mechanical features and durability of concrete incorporating recycled coarse aggregate and nano-silica: experimental study, prediction, and optimization. J. Build. Eng..

[45] Liu X., Xie X., Liu R., Lyu K., Zuo J., Li S., Liu L., Shah S.P. (2023). Research on the durability of nano-SiO2 and sodium silicate Co-modified recycled coarse aggregate (RCA) concrete. Construct. Build. Mater..

[46] Han Y., Lin R., Wang X.-Y. (2023). Preparation of nano calcite by the carbon capture technology to improve the performance of ultrahigh-performance concrete containing calcined clay. ACS Sustain Chem Eng.

[47] ElZein B., Elrashidi A., Dogheche E., Jabbour G. (2022). Analyzing the mechanism of zinc oxide nanowires bending and bundling induced by Electron beam under scanning Electron microscope using numerical and simulation analysis. Materials.

[48] Emara A., Yousef A., ElZein B., Jabbour G., Elrashidi A. (2022). Enhanced broadband metamaterial absorber using plasmonic nanorods and muti-dielectric layers based on ZnO substrate in the frequency range from 100 GHz to 1000 GHz. Crystals.

[49] Elzein B., Yao Y., Barham A.S., Dogheche E., Jabbour G.E. (2020). Toward the growth of self-catalyzed zno nanowires perpendicular to the surface of silicon and glass substrates, by pulsed laser deposition. Materials.

[50] ElZein B., Salah N., Barham A.S., Elrashidi A., Al Khatab M., Jabbour G. (2023). Influence of temperature on the growth of vertically aligned ZnO nanowires in wet oxygen environment. Crystals.

[51] El Zein B., Habib S.S. (2009).

[52] El Zein B. (2013). Self-sufficient energy harvesting in robots using nanotechnology. J Adv Robot Automat.

[53] Bhattacharjee S., Girigoswami A., Nag M., Rubab T., Girigoswami K. (2023). Role of nanotechnology as a zero waste tool. Environ. Qual. Manag..

[54] Kunduru K.R., Nazarkovsky M., Farah S., Pawar R.P., Basu A., Domb A.J. (2017). Water Purification.

[55] Nie C., Li X., Shi S., Wang Y., Lyu X., Li G., Zhu X., Wang Z. (2023). Eco-friendly strategy for advanced recycling waste copper from spent lithium-ion batteries: preparation of micro-nano copper powder. Sep. Purif. Technol..

[56] Linley S., Thomson N.R. (2021). Environmental applications of nanotechnology: nano-enabled remediation processes in water, soil and air treatment. Water Air Soil Pollut..

[57] Singha I., Kumar Mishrab P. (2020). Nano-membrane filtration a novel application of nanotechnology for waste water treatment. Mater Today Proc.

[58] Savage N., Diallo M.S. (2005). Nanomaterials and water purification: opportunities and challenges. J. Nanoparticle Res..

[59] Das S.K., Khan Md M.R., Guha A.K., Das A.R., Mandal A.B. (2012). Silver-nano biohybride material: synthesis, characterization and application in water purification. Bioresour. Technol..

[60] Nguyen L.H., Nguyen B.-S., Le D.-T., Alomar T.S., AlMasoud N., Ghotekar S., Oza R., Raizada P., Singh P., Nguyen V.-H. (2023). A concept for the biotechnological minimizing of emerging plastics, micro- and nano-plastics pollutants from the environment: a review. Environ. Res..

[61] Goh P.S., Ismail A.F., Hilal N. (2016). Nano-enabled membranes technology: sustainable and revolutionary solutions for membrane desalination?. Desalination.

[62] Bhoj Y., Pandey G., Bhoj A., Tharmavaram M., Rawtani D. (2021). Recent advancements in practices related to desalination by means of nanotechnology. Chemical Physics Impact.

[63] Kaviti A.K., Ram A.S., Aruna Kumari A., Hussain S. (2021). A brief review on high-performance nano materials in solar desalination. Mater Today Proc.

[64] Wei H., Zhao S., Zhang X., Wen B., Su Z. (2021). The future of freshwater access: functional material-based nano-membranes for desalination. Mater. Today Energy.

[65] El Zein B., Yao Y., Boulfrad S., Jabbour G., Dogheche E. (2013).

[66] Elrashidi A., Emara A., Yousef A., Elzein B. (2019). Light harvesting improvement of A-Si:H solar cell through nano-grating structure and plasmonic nanoparticles. J. Nanoelectron. Optoelectron..

[67] Saboor F.H., Nguyen T.A. (2022). Nanotechnology in Fuel Cells.

[68] Asghar M.I., Lund P.D. (2016). Improving catalyst stability in nano-structured solar and fuel cells. Catal. Today.

[69] Rahman M.A., Wang X.J., Wen C.E. (2013). High energy density metal-air batteries: a review. J. Electrochem. Soc..

[70] McDowell M.T. (2021). The role of nanoscale science for advancing batteries. Nano Lett..

[71] Hilder M., Winther-Jensen B., Clark N.B. (2009). Paper-based, printed zinc-air battery. J. Power Sources.

[72] Tafete G.A., Abera M.K., Thothadri G. (2022). Review on nanocellulose-based materials for supercapacitors applications. J. Energy Storage.

[73] Deshmukh P.R., Pusawale S.N., Jagadale A.D., Lokhande C.D. (2012). Supercapacitive performance of hydrous ruthenium oxide (RuO2 center dot NH(2)O) thin films deposited by SILAR method. J. Mater. Sci..

[74] Dubal D.P., Holze R. (2013). A successive ionic layer adsorption and reaction (SILAR) method to induce Mn3O4 nanospots on CNTs for supercapacitors. New J. Chem..

[75] Chang S.P., Wen C.H., Chang S.J. (2014). Two-dimensional ZnO Nanowalls for gas sensor and photoelectrochemical applications. Electron. Mater. Lett..

[76] Chen T.P., Chang S.P., Hung F.Y., Chang S.J., Hu Z.S., Chen K.J. (2013). Simple fabrication process for 2D ZnO Nanowalls and their potential application as a methane sensor. Sensors.

[77] Elrashidi A. (2016). Investigating the performance of ultra-sensitive optical sensor using plasmonic nanoparticles. Nanosci. Nanotechnol. Lett..

[78] Wang Q., Li L., Hong Y., Zhai Q., He Y. (2023). Novel insights into indoor air purification capability of microalgae: characterization using multiple air quality parameters and comparison with common methods. Environ. Sci. Pollut. Control Ser..

[79] Thakur A., Kumar A. (2022). Nanotechnology in the Automotive Industry.

[80] Werner M., Wondrak W., Johnston C. (2018). Nanoscience and Nanotechnology: Advances and Developments in Nano-Sized Materials.

[81] Dhinakaran V., Shree M.V. (2021). Nanomaterials and Nanocomposites.

[82] Selvaraj S.K., Ramesh R., Narendhra T.M.V., Agarwal I.N., Chadha U., Paramasivam V., Palanisamy P. (2021). New developments in carbon-based nanomaterials for automotive brake pad applications and future challenges. J. Nanomater..

[83] Shafique M., Luo X. (2019). Nanotechnology in transportation vehicles: an overview of its applications, environmental, health and safety concerns. Materials.

[84] Xie X., Hao C., Huang Y., Huang Z. (2020). Influence of TiO2-based photocatalytic coating road on traffic-related NOx pollutants in urban street canyon by CFD modeling. Sci. Total Environ..

[85] Malik S., Muhammad K., Waheed Y. (2023). Nanotechnology: a revolution in modern industry. Molecules.

[86] Abdel-Fatah M.A. (2018). Nanofiltration systems and applications in wastewater treatment: review article. Ain Shams Eng. J..

[87] Usman M., Farooq M., Wakeel A., Nawaz A., Cheema S.A., Rehman H. ur, Ashraf I., Sanaullah M. (2020). Nanotechnology in agriculture: current status, challenges and future opportunities. Sci. Total Environ..

[88] Ali M.A., Rehman I., Iqbal A., Din S., Rao A.Q., Latif A., Samiullah T., Azam S., Husnain T. (2014). Nanotechnology : a new frontier in agriculture. International Journal Advancements in Life Sciences.

[89] Periakaruppan R., Romanovski V., Thirumalaisamy S.K., Palanimuthu V., Sampath M.P., Anilkumar A., Sivaraj D.K., Ahamed N.A.N., Murugesan S., Chandrasekar D., Selvaraj K.S.V. (2023). Innovations in modern nanotechnology for the sustainable production of agriculture. ChemEngineering.

[90] Shelar A., Nile S.H., Singh A.V., Rothenstein D., Bill J., Xiao J., Chaskar M., Kai G., Patil R. (2023). Recent advances in nano-enabled seed treatment strategies for sustainable agriculture: challenges, risk assessment, and future perspectives. Nano-Micro Lett..

[91] Aamir Iqbal M. (2020). Sustainable Crop Production.

[92] Singh S.P., Keswani C., Minkina T., Ortiz A., Sansinenea E. (2023). Nano-inputs: a next-generation solution for sustainable crop production. J. Plant Growth Regul..

[93] Akinhanmi F.O., Ayanda O.I., Ahuekwe E.F., Dedeke G.A. (2023). Mitigating the impacts of the COVID-19 pandemic on crop farming: a nanotechnological approach. Agriculture.

[94] Bayat M., Pakina E., Astarkhanova T., Sediqi A.N., Zargar M., Vvedenskiy V. (2019). Review on agro-nanotechnology for ameliorating strawberry cultivation. Research on Crops.

[95] Li P., Zeng H.C. (2017). Hierarchical nanocomposite by the integration of reduced graphene oxide and amorphous carbon with ultrafine MgO nanocrystallites for enhanced CO2 capture. Environ. Sci. Technol..

[96] Tahir M., Amin N.A.S. (2017). Photo-induced CO2 reduction by hydrogen for selective CO evolution in a dynamic monolith photoreactor loaded with Ag-modified TiO2 nanocatalyst. Int. J. Hydrogen Energy.

[97] (2017). Nano-zeolite amendment to minimize greenhouse gas emission in rice soil. JOURNAL OF ENVIRONMENTAL NANOTECHNOLOGY.

[98] Selimefendigil F., Sirin C., Ghachem K., Kolsi L., Alqahtani T., Algarni S. (2022). Enhancing the performance of a greenhouse drying system by using triple-flow solar air collector with nano-enhanced absorber coating. Case Stud. Therm. Eng..

[99] Kong F., Wang J., Hou W., Cui Y., Yu L., Zhang Y., Wang S. (2023). Influence of modified biochar supported sulfidation of nano-zero-valent-iron (S-nzvi/BC) on nitrate removal and greenhouse gas emission in constructed wetland. J. Environ. Sci. (China).

[100] Li J., Li H.B., Xue Y., Fang H.L., Wang W. (2014). Facile electrodeposition of environment-friendly Cu2O/ZnO heteroj unction for robust photoelectrochemical biosensing. Sensors and Actuators B-Chemical.

[101] Al-Obaidi J.R., Yahya Allawi M., Salim Al-Taie B., Alobaidi K.H., Al-Khayri J.M., Abdullah S., Ahmad-Kamil E.I. (2022). The environmental, economic, and social development impact of desertification in Iraq: a review on desertification control measures and mitigation strategies. Environ. Monit. Assess..

[102] Biswas P., Polash S.A., Dey D., Kaium Md A., Mahmud A.R., Yasmin F., Baral S.K., Islam Md A., Rahaman T.I., Abdullah A., Ema T.I., Khan D.A., Bibi S., Chopra H., Kamel M., Najda A., Fouda M.M.A., Rehan U.M., Mheidat M., Alsaidalani R., Abdel-Daim M.M., Hasan Md N. (2023). Advanced implications of nanotechnology in disease control and environmental perspectives. Biomed. Pharmacother..

[103] Lavigne Philippot M., Costa D., Cardellini G., De Sutter L., Smekens J., Van Mierlo J., Messagie M. (2023). Life cycle assessment of a lithium-ion battery with a silicon anode for electric vehicles. J. Energy Storage.

[104] Nandal N., Prajapati P.K., Abraham B.M., Jain S.L. (2022). CO2 to ethanol: a selective photoelectrochemical conversion using a ternary composite consisting of graphene oxide/copper oxide and a copper-based metal-organic framework. Electrochim. Acta.

[105] Tai Y., Liang H., Zaki A., El Hadri N., Abshaev A.M., Huchunaev B.M., Griffiths S., Jouiad M., Zou L. (2017). Core/shell microstructure induced synergistic effect for efficient water-droplet formation and cloud-seeding application. ACS Nano.

[106] Wang X., Yu J., Sun G., Ding B. (2016). Electrospun nanofibrous materials: a versatile medium for effective oil/water separation. Mater. Today.

[107] Pham L.Q., Olekhnovich R.O., Uspenskaya M.V., Vu T.H.N. (2021). Study on polyvinyl chloride nanofibers ability for oil spill elimination. Iran. Polym. J. (Engl. Ed.).

[108] Huang L., Song F., Ding H., Wang Y., Zhu W. (2022). Hydrophobic polyacrylonitrile/pitch electrospun nanofibers for oil spill cleanup: fabrication, optimization, and kinetic investigations. J. Water Proc. Eng..

[109] Li X., Yang Z., Peng Y., Zhang F., Lin M., Zhang J., Lv Q., Dong Z. (2022). Self-powered aligned porous superhydrophobic sponge for selective and efficient absorption of highly viscous spilled oil. J. Hazard Mater..

[110] Mitchell M.J., Billingsley M.M., Haley R.M., Wechsler M.E., Peppas N.A., Langer R. (2021). Engineering precision nanoparticles for drug delivery. Nat. Rev. Drug Discov..

[111] Roger L., Lewinski N., Putnam H., Chen S., Roxbury D., Tresguerres M., Wangpraseurt D. (2023). Nanotechnology for coral reef conservation, restoration and rehabilitation. Nat. Nanotechnol..

[112] Hasnidawani J.N., Azlina H.N., Norita H., Bonnia N.N. (2018). Hardness and adhesion performances of nanocoating on carbon steel. IOP Conf. Ser. Mater. Sci. Eng..

[113] Bhat I.A. (2023). Nanotechnology in reproduction, breeding and conservation of fish biodiversity: current status and future potential. Rev Aquac.

[114] Kristanti R.A., Liong R.M.Y., Hadibarata T. (2021). Soil remediation applications of nanotechnology. Tropical Aquatic and Soil Pollution.

[115] Bakshi M., Abhilash P.C. (2020). Nano-Materials as Photocatalysts for Degradation of Environmental Pollutants.

[116] Yien R.M.K., Matos A.P., dos S., Gomes A.C.C., Garófalo D. de A., Santos-Oliveira R., Simas N.K., Ricci-Júnior E. (2023). Nanotechnology promoting the development of products from the biodiversity of the asteraceae family. Nutrients.

[117] Kah M. (2015). Nanopesticides and nanofertilizers: emerging contaminants or opportunities for risk mitigation?. Front. Chem..

[118] Singh J., Kumar S., Alok A., Upadhyay S.K., Rawat M., Tsang D.C.W., Bolan N., Kim K.H. (2019). The potential of green synthesized zinc oxide nanoparticles as nutrient source for plant growth. J. Clean. Prod..

[119] De Souza A., Govea-Alcaide E., Masunaga S.H., Fajardo-Rosabal L., Effenberger F., Rossi L.M., Jardim R.F. (2019). Impact of Fe3O4 nanoparticle on nutrient accumulation in common bean plants grown in soil. SN Appl. Sci..

[120] Vijai Anand K., Reshma M., Kannan M., Muthamil Selvan S., Chaturvedi S., Shalan A.E., Govindaraju K. (2021). Preparation and characterization of calcium oxide nanoparticles from marine Molluscan shell waste as nutrient source for plant growth. J Nanostructure Chem.

[121] Shafiq F., Anwar S., Firdaus‐e‐Bareen, Zhang L., Ashraf M. (2023). Nano‐biochar: properties and prospects for sustainable agriculture. Land Degrad. Dev..

[122] Jha A., Pathania D., Sonu, Damathia B., Raizada P., Rustagi S., Singh P., Rani G.M., Chaudhary V. (2023). Panorama of biogenic nano-fertilizers: a road to sustainable agriculture. Environ. Res..

[123] Sharma B., Tiwari S., Kumawat K.C., Cardinale M. (2023). Nano-biofertilizers as bio-emerging strategies for sustainable agriculture development: potentiality and their limitations. Sci. Total Environ..

[124] Upadhyay K., katel shambhu, Mandal H.R., Yadav S.P.S., Kharel A., Dahal R. (2021). Nanotechnology for agricultural transformation: a review. Fundamental and Applied Agriculture.

[125] Jadhav R., Pawar P., Choudhari V., Topare N., Raut-Jadhav S., Bokil S., Khan A. (2023). An overview of antimicrobial nanoparticles for food preservation. Mater Today Proc.

[126] Sahoo M., Vishwakarma S., Panigrahi C., Kumar J. (2021). Nanotechnology: current applications and future scope in food. Food Frontiers.

[127] Saini P., Kamalesu Lalita (2023). Manikanika. Review on nanotechnology “impact on the food services industry.”. Mater Today Proc.

[128] Priyanka S., S K.R.N., R S.A.B., John A. (2023). Biocompatible green technology principles for the fabrication of food packaging material with noteworthy mechanical and antimicrobial properties-- A sustainable developmental goal towards the effective, safe food preservation strategy. Chemosphere.

[129] Zhang W., Ahari H., Zhang Z., Jafari S.M. (2023). Role of silica (SiO2) nano/micro-particles in the functionality of degradable packaging films/coatings and their application in food preservation. Trends Food Sci. Technol..

[130] Gabor F. (2011). Characterization of nanoparticles intended for drug delivery. Sci. Pharm..

[131] Sim S., Wong N. (2021). Nanotechnology and its use in imaging and drug delivery. Biomed Rep.

[132] Shi J., Votruba A.R., Farokhzad O.C., Langer R. (2010). Nanotechnology in drug delivery and tissue engineering: from discovery to applications. Nano Lett..

[133] Deng Y., Zhang X., Shen H., He Q., Wu Z., Liao W., Yuan M. (2020). Application of the nano-drug delivery system in treatment of cardiovascular diseases. Front. Bioeng. Biotechnol..

[134] Wang S., Xu T., Yang Y., Shao Z. (2015). Colloidal stability of silk fibroin nanoparticles coated with cationic polymer for effective drug delivery. ACS Appl. Mater. Interfaces.

[135] Bardhan N. (2022). Nanomaterials in diagnostics, imaging and delivery: applications from COVID-19 to cancer. MRS Commun.

[136] Thwala L.N., Ndlovu S.C., Mpofu K.T., Lugongolo M.Y., Mthunzi-Kufa P. (2023). Nanotechnology-based diagnostics for diseases prevalent in developing countries: current advances in point-of-care tests. Nanomaterials. MDPI April.

[137] Singh A., Amiji M.M. (2022). Application of nanotechnology in medical diagnosis and imaging. Curr. Opin. Biotechnol..

[138] Danney, R. Journal of Nanomedicine & Biotherapeutic Discovery Nanotechnology for Revolutionizing Medical Imagining for Accurate and Precise Disease Detection The Power of Nanotechnology. J Nanomedicine Biotherapeutic Discov 13, 1000204. 10.4172/2155-983X.23.13.204.

[139] Muller D.A., Nakagawa N., Ohtomo A., Grazul J.L., Hwang H.Y. (2004). Atomic-scale imaging of nanoengineered oxygen vacancy profiles in SrTiO3. Nature.

[140] Amaral M., do Vale F., Silva J., Caramelo F., Veiga G. (2014). In vitro zinc-air battery evaluation for use in intraoral medical devices. Journal of Medical Devices-Transactions of the Asme.

[141] Harrison R.H., St-Pierre J.P., Stevens M.M. (2014). Tissue engineering and regenerative medicine: a year in review. Tissue Engineering - Part B: Reviews.

[142] Zhang S., Chen X., Shan M., Hao Z., Zhang X., Meng L., Zhai Z., Zhang L., Liu X., Wang X. (2023). Convergence of 3D bioprinting and nanotechnology in tissue engineering scaffolds. Biomimetics.

[143] Rahmani Del Bakhshayesh A., Saghebasl S., Asadi N., Kashani E., Mehdipour A., Nezami Asl A., Akbarzadeh A. (2023). Recent advances in nano‐scaffolds for tissue engineering applications: toward natural therapeutics. WIREs Nanomedicine and Nanobiotechnology.

[144] Gomes M.E., Rodrigues M.T., Domingues R.M.A., Reis R.L. (2017). Tissue engineering and regenerative medicine: new trends and directions - a year in review. Tissue Engineering - Part B: Reviews.

[145] Asghar M.S., Li J., Ahmed I., Ghazanfar U., Irshad M.S., Idrees M., Haq Z., Rizwan M., Sheikh F., Yasmeen F. (2021). Antioxidant, and enhanced flexible nano porous scaffolds for bone tissue engineering applications. Nano Select.

[146] Adel I.M., Elmeligy M.F., Elkasabgy N.A. (2022). Conventional and recent trends of scaffolds fabrication: a superior mode for tissue engineering. Pharmaceutics.

[147] Garalleh H., Thamwattana N., Cox B.J., Hill J.M. (2016). Encapsulation of L-histidine amino acid inside single-walled carbon nanotubes. J Biomater Tissue Eng.

[148] Ho S.T., Hutmacher D.W. (2006). A comparison of micro CT with other techniques used in the characterization of scaffolds. Biomaterials.

[149] Lozano G., Rodriguez S.R.K., Verschuuren M.A., Rivas J.G. (2016). Metallic nanostructures for efficient LED lighting. Light Sci. Appl..

[150] Bezshlyakh D.D., Spende H., Weimann T., Hinze P., Bornemann S., Gülink J., Canals J., Prades J.D., Dieguez A., Waag A. (2020). Directly addressable GaN-based nano-LED arrays: fabrication and electro-optical characterization. Microsyst Nanoeng.

[151] Rogers D.J., Sandana V.E., Teherani F.H., Razeghi M., Drouhin H.J. (2009). Fabrication of nanostructured heterojunction LEDs using self-forming “moth-eye” type arrays of n-ZnO nanocones grown on p-Si (111) substrates by pulsed laser deposition. Zinc Oxide Materials and Devices Iv.

[152] Dai X., Messanvi A., Zhang H., Durand C., Eymery J., Bougerol C., Julien F.H., Tchernycheva M. (2015). Flexible light-emitting diodes based on vertical nitride nanowires. Nano Lett..

[153] Kamali-Sarvestani R., Weber P., Clayton M., Meyers M., Slade S. (2020). Virtual reality to improve nanotechnology education: development methods and example applications. IEEE Nanotechnol Mag.

[154] Jones M.G., Gardner G.E., Falvo M., Taylor A. (2015). Precollege nanotechnology education: a different kind of thinking. Nanotechnol. Rev..

[155] Ghattas N.I., Carver J.S. (2012). Integrating nanotechnology into school education: a review of the literature. Res. Sci. Technol. Educ..

[156] Şenel Zor T., Aslan O. (2018). The effect of activity-based nanoscience and nanotechnology education on pre-service science teachers' conceptual understanding. J. Nanoparticle Res..

[157] Praveen K., Prayag G., Satish Y.M., Basavaraj T. (2022). Emerging role of nanotechnology in engineering education. Journal of Engineering Education Transformations.

[158] Mandrikas A., Michailidi E., Stavrou D. (2020). Teaching nanotechnology in primary education. Res. Sci. Technol. Educ..

[159] Mg K., V K., F H. (2015). History and possible uses of nanomedicine based on nanoparticles and nanotechnological progress. J. Nanomed. Nanotechnol..

[160] Bellucci S., Chiaretti M., Onorato P., Rossella F., Grandi M.S., Galinetto P., Sacco I., Micciulla F. (2010). Micro-Raman study of the role of sterilization on carbon nanotubes for biomedical applications. Nanomedicine.

[161] Khalid Iqbal R. (2019). Nanotechnology combating fight against cancer. Annals of Advanced Biomedical Sciences.

[162] Venkatraman P.D., Sayed U., Parte S., Korgaonkar S. (2021). Development of advanced textile finishes using nano-emulsions from herbal extracts for organic cotton fabrics. Coatings.

[163] Bhalla N., Jayaprakash A., Ingle N., Patel H., Patri S.V., Haranath D. (2022). Fabrication and infusion of potent silver doped nano ZnO aimed to advance germicidal efficacy of health and hygiene products. J. Sci.: Advanced Materials and Devices.

[164] Bhalla N., Ingle N., Patel H., Jayaprakash A., Patri S.V., Kaushik A., Haranath D.A. (2022). Facile approach to fabricate and embed multifunctional nano ZnO into soap matrix and liquid cleansing products for enhanced antibacterial and photostability for health and hygiene applications. Arab. J. Chem..

[165] Rajput A., Ramachandran M., Gotmare V.D., Raichurkar P.P. (2017). Recent bioactive materials for development of eco-friendly dippers: an overview. J. Pharmaceut. Sci. Res..

[166] Kong Xiang-Ping, Zhang Bao-Hua, Wang Juan (2021). Multiple roles of mesoporous silica in safe pesticide application by nanotechnology: a review. J. Agric. Food Chem..

[167] Chandrasekaran R., Periakaruppan R. (2023). Nanometal Oxides in Horticulture and Agronomy.

[168] Adisa I.O., Pullagurala V.L.R., Peralta-Videa J.R., Dimkpa C.O., Elmer W.H., Gardea-Torresdey J.L., White J.C. (2019). Recent advances in nano-enabled fertilizers and pesticides: a critical review of mechanisms of action. Environ. Sci.: Nano.

[169] Manna S., Roy S., Dolai A., Ravula A.R., Perumal V., Das A. (2023). Current and future prospects of “all-organic” nanoinsecticides for agricultural insect pest management. Frontiers in Nanotechnology..

[170] Selim M.S., Shenashen M.A., Elmarakbi A., Fatthallah N.A., Hasegawa S. ichi, El- Safty S.A. (2017). Synthesis of ultrahydrophobic and thermally stable inorganic–organic nanocomposites for self-cleaning foul release coatings. Chem. Eng. J..

[171] Sui M.H., Zhang L.D., Sheng L., Huang S.H., She L. (2013). Synthesis of ZnO coated multi-walled carbon nanotubes and their antibacterial activities. Sci. Total Environ..

[172] Bowman D.M. (2017). More than a decade on: mapping today's regulatory and policy landscapes following the publication of nanoscience and nanotechnologies: opportunities and uncertainties. Nanoethics.

[173] Dowling a, Clift R., Grobert N., Hutton D., Oliver R., O’neill O., Pethica J., Pidgeon N., Porritt J., Ryan J. (2004). Nanoscience and nanotechnologies : opportunities and uncertainties. London The Royal Society The Royal Academy of Engineering Report.

[174] Roco M.C. (2017). Nanotechnology Commercialization.

[175] Zein B.E.L., Jarwan, Al A. (2021). Paths to a culture of tolerance and peace.

[176] Abbasi A.S., Rehman R.A., Anwar A., Mehak U.A. (2023).

[177] Akhtar S., Rao D., Din S.U. (2023).

[178] Rahmat M., Naz S., Kiran S. (2023). Nano-Trackers (Nano-Sensors) for Forensics Investigation.

[179] Rajendran J., Karri R., Wendt J.B., Potkonjak M., McDonald N., Rose G.S., Wysocki B. (2015). Nano meets security: exploring nanoelectronic devices for security applications. Proc. IEEE.

[180] Dressler F., Kargl F. (2012). Towards security in nano-communication: challenges and opportunities. Nano Commun Netw.

[181] Alabdulatif A., Thilakarathne N.N., Lawal Z.K., Fahim K.E., Zakari R.Y. (2023). Internet of nano-things (IoNT): a comprehensive review from architecture to security and privacy challenges. Sensors.

[182] Atlam H.F., Walters R.J., Wills G.B. (2018). Proceedings of the 2018 2nd International Conference on Cloud and Big Data Computing.

[183] Agarwal K.K., Agarwal K.K., Agarwal S. (2017). Evolution of Internet of nano Things (IoNT). International Journal of Engineering Technology Science and Research.

[184] Senesac L., Thundat T.G. (2008). Nanosensors for trace explosive detection. Mater. Today.

[185] Liu Y., Chen C.L., Zhang Y., Sonkusale S.R., Wang M.L., Dokmeci M.R. (2013). SWNT based nanosensors for wireless detection of explosives and chemical warfare agents. IEEE Sens J.

[186] Adegoke O., Daeid N.N. (2021). Colorimetric optical nanosensors for trace explosive detection using metal nanoparticles: advances, pitfalls, and future perspective. Emerging Topics in Life Sciences.

[187] Choi I., Kim J.G., Seo I.S., Lee D.G. (2012). Radar absorbing sandwich construction composed of CNT, PMI foam and carbon/epoxy composite. Compos. Struct..

[188] Zaki B.A.R., Rashid F.L., Al Baiati M.N. (2022). Synthesis and characterization of a novel nano-composite material for microwave absorption. International Journal on Technical and Physical Problems of Engineering.

[189] Ramezani H., Abohorlu Doğramaci P. (2021). The impact of using nano self-healing concrete in flexible houses. Future Cities and Environment.

[190] Liu Y., Zhang H., Zhang Y., Liang C., An Q. (2022). Rendering passive radiative cooling capability to cotton textile by an alginate/CaCO3 coating via synergistic light manipulation and high water permeation. Compos. B Eng..

[191] Du Y., Kim H.E. (2022). Research trends of the application of aerogel materials in clothing. Fashion and Textiles.

[192] Dolez P.I., Marsha S., McQueen R.H. (2022). Fibers and textiles for personal protective equipment: review of recent progress and perspectives on future developments. Textiles.

[193] Cui X. (2021). Preparation and characterization of polyimide/TiO2 nano-composites for non-lethal weapon. J. Phys. Conf..

[194] (2017). Nanotechnology commercialization: manufacturing processes and products. Focus Catal..

[195] Yildirim M., Candan Z. (2023). Advanced Renewable Nanomaterials for Sustainable Development.

[196] Prasad R., Bhattacharyya A., Nguyen Q.D. (2017). Nanotechnology in sustainable agriculture: recent developments, challenges, and perspectives. Front. Microbiol..

[197] Assey G.E., Malasi W.S. (2021). Advances in nanomaterials sciences and nanotechnology for sustainable development: a review. Tanzan. J. Sci..

[198] Bhattacharya B., Roy P., Bhattacharya S., Prasad B., Mandal A.K. (2022). Engineered Nanomaterials for Sustainable Agricultural Production, Soil Improvement and Stress Management.

[199] Mangematin V., Walsh S. (2012). The future of nanotechnologies. Technovation.

[200] Nagy J.B. (2021). The past, the present and the future of nanotechnologies. Acta Materialia Transylvanica.

[201] Aithal P.S., Aithal S. (2022). Sustainable Nanotechnology: Strategies, Products, and Applications.

[202] Al-Obaidy G.S., Abdulrahman M.F., Alobaidy B.S.J. (2023). Future trends for green environmental applications of nanotechnology: a review. Anbar Journal of Agricultural Sciences.

[203] Bertolotti M., Tahir Muhammad Bilal (2021). Muhammad Rafique and Muhammad Sagir.

[204] Li S., Chen L., Fu Y. (2023). Nanotechnology-based ocular drug delivery systems: recent advances and future prospects. J. Nanobiotechnol..

[205] Sobianin I., Psoma S.D., Tourlidakis A. (2022). Recent advances in energy harvesting from the human body for biomedical applications. Energies.

[206] Ahmed A., Azam A., Wang Y., Zhang Z., Li N., Jia C., Mushtaq R.T., Rehman M., Gueye T., Shahid M.B., Wajid B.A. (2021). Additively manufactured nano-mechanical energy harvesting systems: advancements, potential applications, challenges and future perspectives. Nano Convergence.

[207] Iavicoli I., Leso V., Ricciardi W., Hodson L.L., Hoover M.D. (2014). Opportunities and challenges of nanotechnology in the green economy. Environmental Health: A Global Access Science Source.

[208] Pandey G., Jain P. (2020).

[209] Gehrke I., Geiser A., Somborn-Schulz A. (2015). Innovations in nanotechnology for water treatment. Nanotechnol. Sci. Appl..

[210] Yao D., Chen Z., Zhao K., Yang Q., Zhang W. (2013). Limitation and challenge faced to the researches on environmental risk of nanotechnology. Procedia Environ Sci.

[211] Omidian H., Mfoafo K. (2023). Exploring the potential of nanotechnology in pediatric healthcare: advances, challenges, and future directions. Pharmaceutics.

[212] Bishoge O.K., Zhang L., Suntu S.L., Jin H., Abraham A., Qi Z. (2018). Toxic/hazardous substances and environmental engineering remediation of water and wastewater by using engineered nanomaterials: a review. Journal of Environmental Science and Health, Part A.

[213] Powell M.C., Griffin M.P.A., Tai S. (2008). Bottom-up risk regulation? How nanotechnology risk knowledge gaps challenge federal and state environmental agencies. Environ. Manag..

[214] A Review on Nanotechnology (2022). Applications in food industry, future opportunities, challenges and potential risks. Journal of Nanotechnology and Nanomaterials.

[215] Fadel T.R., Steevens J.A., Thomas T.A., Linkov I. (2015). The challenges of nanotechnology risk management. Nano Today.

[216] Resnik D.B., Tinkle S.S. (2007). Ethical issues in clinical trials involving nanomedicine. Contemp. Clin. Trials.

[217] Allon I., Ben-Yehudah A., Dekel R., Solbakk J.H., Weltring K.M., Siegal G. (2017). Ethical issues in nanomedicine: tempest in a teapot?. Med Health Care Philos.

[218] Jones M.G., Blonder R., Gardner G.E., Albe V., Falvo M., Chevrier J. (2013). Nanotechnology and nanoscale science: educational challenges. Int. J. Sci. Educ..

[219] Jemala M. (2022). Systemic technology innovation management and analysis of other forms of IP protection. International Journal of Innovation Studies.

[220] Siri J.G.S., Fernando C.A.N., De Silva S.N.T. (2020). Nanotechnology and protection of intellectual property: emerging trends. Recent Pat. Nanotechnol..

